# The pseudogene GBP1P1 suppresses influenza A virus replication by acting as a protein decoy for DHX9

**DOI:** 10.1128/jvi.00738-24

**Published:** 2024-06-28

**Authors:** Xiaohang Yu, Jiaxin Tian, Yihe Wang, Ning Su, Jinna Luo, Ming Duan, Ning Shi

**Affiliations:** 1 State Key Laboratory for Diagnosis and Treatment of Severe Zoonotic Infectious Diseases, Key Laboratory for Zoonosis Research of the Ministry of Education, Institute of Zoonosis, College of Veterinary Medicine, Jilin University, Changchun, Jilin Province, China; 2 School of Life Science and Technology, Changchun University of Science and Technology, Changchun, Jilin Province, China; Emory University School of Medicine, Atlanta, Georgia, USA

**Keywords:** influenza A virus, pseudogene, long noncoding RNA, GBP1P1, DHX9, replication

## Abstract

Recently, substantial evidence has demonstrated that pseudogene-derived long noncoding RNAs (lncRNAs) as regulatory RNAs have been implicated in basic physiological processes and disease development through multiple modes of functional interaction with DNA, RNA, and proteins. Here, we report an important role for GBP1P1, the pseudogene of guanylate-binding protein 1, in regulating influenza A virus (IAV) replication in A549 cells. GBP1P1 was dramatically upregulated after IAV infection, which is controlled by JAK/STAT signaling. Functionally, ectopic expression of GBP1P1 in A549 cells resulted in significant suppression of IAV replication. Conversely, silencing GBP1P1 facilitated IAV replication and virus production, suggesting that GBP1P1 is one of the interferon-inducible antiviral effectors. Mechanistically, GBP1P1 is localized in the cytoplasm and functions as a sponge to trap DHX9 (DExH-box helicase 9), which subsequently restricts IAV replication. Together, these studies demonstrate that GBP1P1 plays an important role in antagonizing IAV replication.

**IMPORTANCE:**

Long noncoding RNAs (lncRNAs) are extensively expressed in mammalian cells and play a crucial role as regulators in various biological processes. A growing body of evidence suggests that host-encoded lncRNAs are important regulators involved in host–virus interactions. Here, we define a novel function of GBP1P1 as a decoy to compete with viral mRNAs for DHX9 binding. We demonstrate that GBP1P1 induction by IAV is mediated by JAK/STAT activation. In addition, GBP1P1 has the ability to inhibit IAV replication. Importantly, we reveal that GBP1P1 acts as a decoy to bind and titrate DHX9 away from viral mRNAs, thereby attenuating virus production. This study provides new insight into the role of a previously uncharacterized GBP1P1, a pseudogene-derived lncRNA, in the host antiviral process and a further understanding of the complex GBP network.

## INTRODUCTION

The term “pseudogene” was first used in 1977 to describe a DNA sequence that was directly adjacent to, and closely resembling, the 5S ribosomal RNA (rRNA) gene in the *Xenopus laevis* genome (1). Since then, a large number of pseudogenes have been discovered in organisms from prokaryotes to eukaryotes. In the last decade, using large-scale sequencing techniques and bioinformatics methods, Encyclopedia of DNA Elements and Functional Annotation of Mammals projects have identified thousands of pseudogene sequences in the human genome (2–4). GENCODE, which currently serves as the most definitive database of annotated pseudogenes, reports a total of 14,737 human pseudogenes (version 43, August 2022, GRCh38.p13).

Traditionally, pseudogenes have been defined as a genomic DNA sequence that looks like a mutated or truncated version of a known functional gene. However, studies over the last few decades have accumulated evidence for the multifaceted functions of pseudogenic DNA, RNA, or protein, among which pseudogene-derived RNA transcripts are the most investigated. Pseudogene-derived transcripts have been considered to be one of the key components of long noncoding RNAs (lncRNAs). Similar to common lncRNAs, pseudogene-derived lncRNAs can also act as critical modulators. For example, an antisense neural nitric oxide synthase (nNOS) pseudogene transcript can hybridize directly to nNOS mRNA and mediate the translational control of nNOS expression (5). A partial retrotranscript pseudogene psi protein phosphatase, Mg^2+^/Mn^2+^ dependent, 1K (ψPPM1K) containing inverted repeats can be processed into two endo-siRNAs that target and repress the expression of the cognate genes *PPM1K* and never in mitosis A-related kinase 8, leading to suppressed hepatocellular carcinoma cell proliferation (6). PTENP1, a pseudogene of phosphatase and tensin homolog (PTEN), regulates cellular levels of PTEN by competitively binding to microRNAs (miRNAs), resulting in the suppression of miRNA-induced silencing (7). Additionally, pseudogene RNAs have the ability to sequester not only miRNAs but also proteins. Lethe, a pseudogene lncRNA, interacts with the NF-κB subunit RelA to inhibit the binding of RelA to DNA and the activation of target genes (8). However, only a small number of pseudogene-derived lncRNAs have been well studied so far.

Guanylate-binding proteins (GBPs), which belong to a large GTPase of the dynamin superfamily, are best known for their diverse activities against invading microbes and pathogens as a part of the innate immune responses (9). Human GBP (hGBP) genes are clustered on chromosome 1 and consist of seven main functional members: hGBP1/2/3/4/5/6/7 (10). hGBP1/2/3/5 have been shown to be involved in restricting viruses by targeting different steps in their life cycle. hGBP1 contributes to the host immune response against influenza A virus (IAV) replication, and hGBP1-mediated antiviral activity is antagonized by the viral nonstructural protein 1 (NS1) via its binding to hGBP1 (11). Similarly, nonstructural (NS) protein 5B of the hepatitis C virus (HCV) can bind directly to hGBP1, thereby blocking its GTPase activity and antiviral activity (12). hGBP-3 has only recently been demonstrated to suppress IAV infection by inhibiting the activity of the viral polymerase complex (13). The antiviral capacity of hGBP2/5 against some enveloped viruses, such as Zika virus (ZIKV), measles virus (MV), human immunodeficiency virus (HIV), respiratory syncytial virus (RSV), and IAV, has also been confirmed (14–16).

Guanylate-binding protein 1 pseudogene 1 (GBP1P1) is also located on chromosome 1 closely adjacent to the GBP gene family. Several studies have shown that the abnormal expression of GBP1P1 is associated with several diseases, such as parenchymal neurocysticercosis, cervical carcinoma, tuberculosis, and hepatocellular carcinoma (17–20). However, the biological functions and underlying mechanisms of GBP1P1 in the pathogenesis of these diseases have not been elucidated. Furthermore, the relationship between GBP1P1 and the influenza A virus has not been documented either. This prompted us to explore the role of GBP1P1 in IAV infection.

In this study, we demonstrated that GBP1P1 is an IAV-induced lncRNA controlled by the JAK/STAT pathway in A549 cells. GBP1P1 significantly inhibits the replication of IAV. Further mechanistic studies reveal that GBP1P1 interacts with DHX9 (DExH-box helicase 9), which promotes IAV replication, and competes with viral mRNA for DHX9 binding. Together, our data identify GBP1P1 as a novel inducible antiviral host factor that acts by as a protein decoy for DHX9.

## MATERIALS AND METHODS

### Cells and cell culture

Human lung cancer A549 and lung adenocarcinoma cell line H1299 cells, human glioblastoma cells (A172), human acute monocytic leukemia cells (THP-1), human neuroblastoma cells (SH-SY5Y), human colon carcinoma cells (Caco-2), Madin–Darby canine kidney cells (MDCK), and human embryonic kidney cells (HEK293T) were maintained in Ham’s F-12K (Kaighn’s) Medium, Dulbecco’s modified Eagle’s medium (Gibco), or RPMI-1640 supplemented with 10% fetal bovine serum (Gibco), penicillin (100 U/ml), and streptomycin (100 µg/mL) at 37°C under 5% CO_2_ atmosphere.

### Virus, viral infection, and virus titer assay

IAV strains including A/Puerto Rico/8/34(H1N1)(PR8), A/WSN/33(H1N1) (WSN), A/Jingfang/86(H1N1), and A/Lufang/30/95(H3N2) were propagated in specific pathogen-free (SPF) embryonated chicken eggs. Unless indicated, cell monolayers were washed and incubated with the virus at a multiplicity of infection (MOI) of 1 for 1 h with a medium containing 2 µg/mL of TPCK (L-1-tosylamido-2-phenylethyl chloromethyl ketone)-treated trypsin (Sigma). After adsorption, the supernatant was aspirated, and cells were cultured with the medium for the indicated times with IAV. Cell culture supernatant was harvested at the indicated time points after virus infection. Virus titers in supernatants after serial dilutions were determined on MDCK cells by 50% tissue-culture infectious dose (TCID_50_) assay.

### Reagents

Ruxolitinib (MedChemExpress), a potent and selective JAK1/2 inhibitor, was used to block JAK/STAT signaling pathway. The polyinosinic-polycytidylic acid (polyI:C)-LMW, CpG oligodeoxynucleotides (CpG-ODN)-2216, and lipopolysaccharide (LPS, from *Escherichia coli* strain K12) were purchased from InvivoGen. Human interferon (IFN)-β recombinant protein (#300–02B.C.BC) was obtained from PeproTech. Ruxolitinib, poly(I:C), CpG-ODN, IFN-β, and LPS at the used concentrations showed no cytotoxic effects on A549 cells. Lipofectamine LTX and RNAi MAX were both from Invitrogen. Antibodies against DHX9 (17721–1-AP) and DEAD Box Protein 1 (DDX1; 11357–1-AP) were both from Proteintech. Anti-IAV M1 antibody was from GeneTex (GTX125928). Anti-IAV NS1 antibody was from Thermo Fisher (PA5-32243). Anti-β-actin antibody was from Bioworld (BS6007M).

### Small interfering RNAs and plasmids

Custom-designed small interfering RNAs (siRNAs) against GBP1P1, DHX9, and siRNA universal negative control #1 (SIC001) were synthesized by Sigma. Sequences of RNA oligos are as follows: 5′-CAUUCAGUGCUUUAUCUAUUU-3′ (sense) and 5′-AUAGAUAAAGCACUGAAUGUU-3′ (antisense) for GBP1P1 and 5′-CAAACCUUGAGCAACGGAAUU-3′ (sense) and 5′-UUCCGUUGCUCAAGGUUUGUU-3′ (antisense) for DHX9. The full length of GBP1P1 and DHX9 were cloned into the pcDNA3(+) vector according to the manufacturer’s protocol.

### Cell stimulation and transient transfections

For stimulation, cells were incubated with LPS, IFN-β, or ruxolitinib at the indicated doses for the indicated times. Transfection of A549 cells with poly(I:C) or CpG-ODN was performed using Lipofectamine LTX (Invitrogen). For plasmid DNA transfection, cells were transiently transfected with 2 µg of plasmids per well of a six-well plate using Lipofectamine LTX. Then, cells were cultured for another 24 h for transient expression. siRNA transfection was conducted with the Lipofectamine RNAiMAX reagent (Invitrogen) according to the manufacturer’s instructions

### Isolation of nuclear and cytoplasmic RNAs

Cells were collected and washed with ice-cold phosphate-buffered saline (PBS) thrice. After centrifugation at 800 *g* for 5 min, supernatants were removed. Cell fractionation was performed to isolate nuclear and cytoplasmic RNAs using a PARIS kit (Invitrogen), following the manufacturer’s protocol.

### Quantitative RT-PCR (qRT-PCR)

Total RNA was purified using TRIzol reagent (Invitrogen) as the manufacturer’s instruction. Equal amounts of RNA (250–1,000 ng) were reverse transcribed with random, oligo(dT) primers, or specific primers using a PrimeScript 1st strand cDNA Synthesis Kit (Takara). Following a previous report, reverse transcription was performed using strand‐ and sense‐specific oligonucleotides for viral RNA (vRNA), complementary RNA (cRNA), and mRNA (oligo dT) (21). For quantitative mRNA and lncRNA expression analysis, comparative real-time PCR was performed using the FastStart Essential DNA Green Master (Roche). Real-time PCR was performed using a LightCycler 96 Real-Time PCR System (Roche), and each assay was run in triplicate. The 2 −ΔΔCt method was used to quantify the relative RNA levels against GAPDH. For RIP-qPCRs, the amount of a target RNA was normalized to 10% total input sample RNA level in each RIP sample and was represented as a percentage relative to the input sample using the delta Ct method. Primers for PCR are listed in Table S1.

### Western blotting

Cells were washed twice with ice-cold PBS and lysed in RIPA buffer containing protease inhibitors (Sigma). For Western blotting, protein samples were denatured at 100°C in SDS-PAGE loading buffer for 10 min and separated by SDS-PAGE. Proteins were transferred onto polyvinylidene difluoride membranes (Millipore) and detected with the indicated antibodies. Membranes were probed with horseradish peroxidase-conjugated goat anti-mouse (Bioworld) or anti-rabbit (Bioworld). Western blotting was developed using ECL chemiluminescent substrate (Pierce), and protein signals were recorded using Gel Doc XR + molecular imager (Bio-Rad).

### Luciferase assay

PathDetect interferon-sensitive response element (ISRE) *cis*-reporting system was purchased from Agilent Technologies. The −3,000 to 0 region of the GBP1P1 gene, which contains five putative STAT1-binding sites, was cloned into the multiple cloning sites of the pGL3-Basic Luciferase vector (Promega). Briefly, A549 cells were cotransfected with the indicated plasmids (firefly luciferase) and pRL-CMV plasmid (renilla luciferase; Promega). After transfection, the cells were infected with IAV and then cultured for 12 h. The cell lysates were assayed for firefly and renilla luciferase activities using the dual-luciferase reporter assay system (Promega) according to the manufacturer’s recommendations. Firefly luciferase activity was normalized to renilla luciferase activity for each transfected well.

### Chromatin immunoprecipitation (ChIP)

A549 cells were infected with PR8 and collected at 12 h post-infection (hpi), and then were subjected to ChIP assays using the Magna ChIP A/G chromatin immunoprecipitation kit (Millipore) following the manufacturer’s instruction. Briefly, the chromatin fraction was immunoprecipitated with 3 µg of anti-STAT1 (Invitrogen; MA5-15071) or IgG control (Millipore) antibody. Chromatin complexes were captured using 20 µL of magnetic beads at 4°C for 4 h or overnight. ChIP-purified DNA was cleaned using PCR purification columns (Qiagen) and subjected to qPCR analysis using primers amplifying genomic regions around the transcription start site (TSS) of indicated regions. Sequences of primers used in ChIP studies are provided in Table S2. The STAT1 enrichment of ChIP samples was normalized to the input DNA and was calculated as 2 −ΔΔCt with normalization against IgG control. Experiments were performed at least three times with independent chromatin samples.

### Fluorescent *in situ* hybridization (FISH)

A549 cells were seeded in dishes with glass bottoms and infected with IAV for 24 h. To detect GBP1P1, FISH assays were performed using Invitrogen FISH Tag Detection Kits according to the manufacturer’s instructions. The template for transcribing anti-sense RNA and sense RNA probes is generated by linearizing the pEASY-T3 vector (TransGen Biotech) expressing GBP1P1. For double FISH, primary and Alexa Fluor secondary antibodies (Invitrogen) were added. The samples were counterstained with 4′,6-diamidino-2-phenylindole (DAPI. Invitrogen) and observed using confocal microscopy.

### RNA pull-down assay and mass spectrometry

Linearized pEASY-T3 vector expressing GBP1P1 was used to synthesize biotinylated GBP1P1 or its antisense control RNA. For transcription *in vitro*, biotin-labeled sense or anti-sense RNAs were synthesized using MAXIscript SP6/T7 Kit (Ambion) and RNA 3′ End Desthiobiotinylation kit (Pierce). RNA pull-downs were performed using the Magnetic RNA-Protein Pull-Down kit (Pierce) according to the manufacturer’s instructions. Protein eluates were resolved on 10% SDS-PAGE and subjected to Silver Staining (Pierce silver stain kit). GBP1P1-specific bands were excised and trypsin digested followed by liquid chromatography with tandem mass spectrometry analysis.

### RNA immunoprecipitation (RIP)

RIP was performed using the Magna RIP RNA-Binding Protein Immunoprecipitation Kit (Millipore) according to the manufacturer’s instructions. In brief, A549 cells with PR8 were collected for RIP at 24 hpi. Cells were lysed in polysome lysis buffer and immunoprecipitated with antibody against DHX9 with protein A/G magnetic beads. The immunoprecipitated RNA was purified using phenol:chloroform:isoamyl alcohol (Invitrogen) and ethanol precipitation, and measured with qRT-PCR. The relative RNA levels were normalized to input, and the fold enrichment was calculated as 2 −ΔΔCt with normalization to IgG control.

### Separation of nuclear and cytoplasmic protein fractions

Nuclear and cytoplasmic proteins were extracted and separated using an NE-PER nuclear and cytoplasmic extraction kit (Pierce). The purity of these fractions was assessed using specific protein markers (GAPDH and Lamin B1).

### Co-immunoprecipitation (Co-IP)

HEK293T cells were transfected with the plasmids indicated and infected with PR8. Cells were lysed with IP lysis buffer at 24 hpi. The lysates were incubated with anti-DHX9 antibody and incubation buffer at 4°C overnight to immunoprecipitate the protein complexes. Meanwhile, control IgG was added to the lysate as a negative control. Protein A Sepharose beads were then added, incubated at 4°C for 4 h, and then eluted with elution buffer. The IP samples were stored at −80°C for western blot analysis.

### Statistical analysis

All data were expressed as the mean ± standard error of the mean (SEM). Means of groups were from at least three independent experiments and compared with Student’s *t*-test (unpaired) or the ANOVA test when appropriate. *P＜0.05 and **P＜0.01 indicated significant difference.

## RESULTS

### GBP1P1 is induced by IAV infection

**Fig 1:**
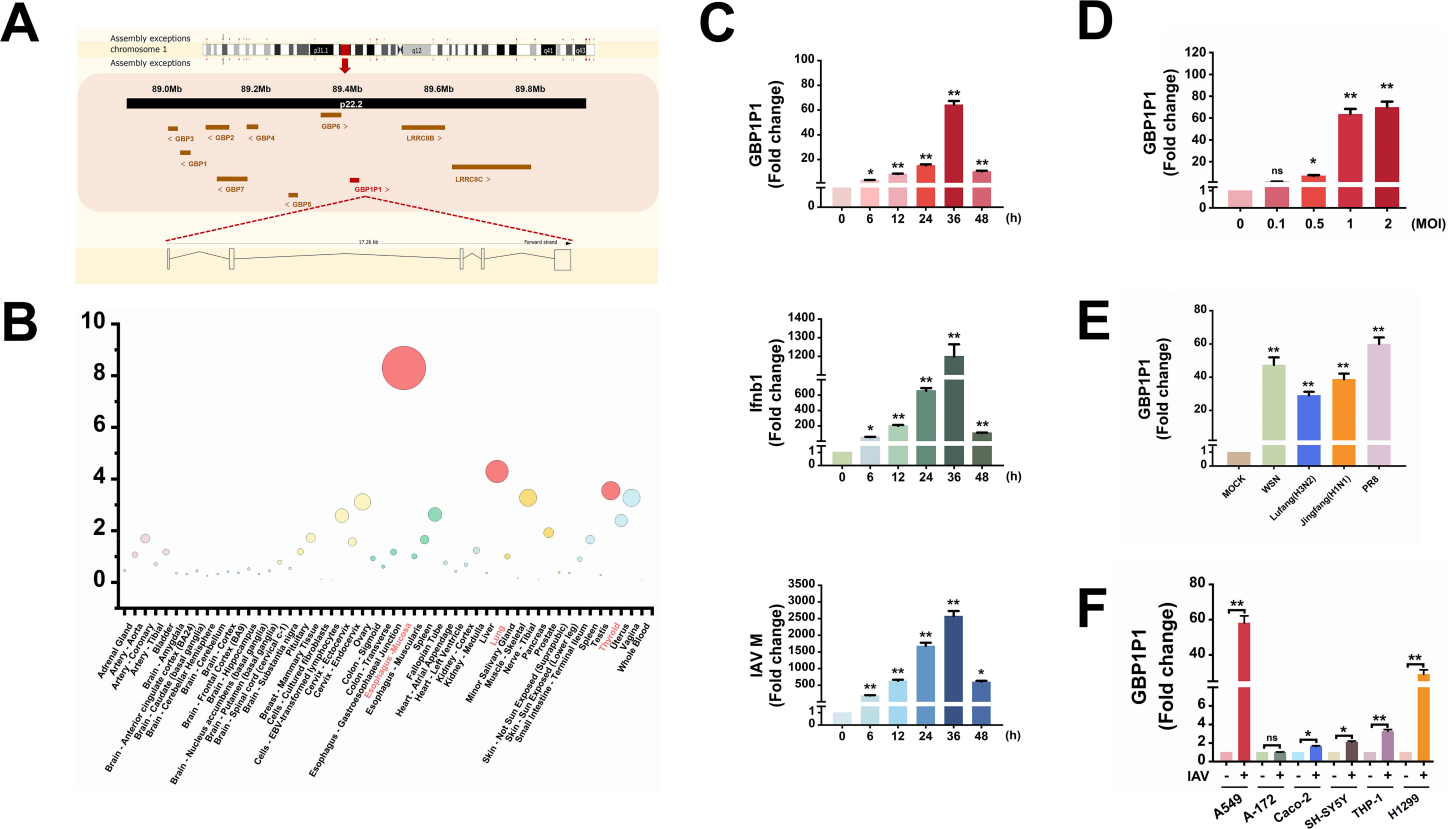
GBP1P1 is Induced by IAV Infection. (**A**) Locus of GBP1P1 gene (labeled with red block) and its nearby coding genes GBP1, GBP2, GBP3, GBP4, GBP5, GBP6, GBP7, LRRC8B, and LRRC8C (labeled with brown) on chromosome 1p22.2 (GRCh38.p14). (**B**) Expression levels of GBP1P1 in different types of tissue using the GTEx database. (**C**) A549 cells were infected with IAV strain H1N1(PR8) for the times indicated. Total RNA was extracted and subjected to qRT-PCR to assess GBP1P1, Ifnb1, and IAV M genes. (**D**) A549 cells were infected with PR8 at indicated MOIs for 24 h. qRT-PCR was performed to determine the GBP1P1 expression. (**E**) The expression of GBP1P1 in A549 cells infected with four IAV strains for 24 hours was measured by qRT-PCR. (**F**) The GBP1P1 expression in indicated human cell lines infected with/without PR8 for 24 h was examined by qRT-PCR. IAV means the PR8 strain unless otherwise specified. All shown qRT-PCRs are representative from three independent experiments with similar results. Results were normalized to GAPDH. Data are shown as means ± SEM. NS, not significant. *P* < 0.05; **P < 0.01 vs 0 hpi or mock-treated cells.

The human pseudogene GBP1P1 is located on chromosome 1p22.2 and mapped to 89, 407, 679–89, 424, 934 forward strand in GRCh38.p14 (Fig. 1A). A cross-species comparative analysis between chicken, mouse, and human revealed that GBP1P1 is present only in the human genome. The analysis of the theoretical coding potential using the Coding Potential Calculator 2 (CPC2) program (http://cpc2.cbi.pku.edu.cn/) shows that the coding probability of GBP1P1 is 0.109819, in a range similar to that obtained for the validated lncRNA HOTAIR (0.184882) and NRAV (0.0990541) and much lower than that of the nearby coding genes GBP1 (1) and leucine-rich repeat-containing 8 VRAC subunit B (LRRC8B) (1), indicating the noncoding potential of GBP1P1. The expression profiles of GBP1P1 from GTEx show that high levels of GBP1P1 can be observed in the esophagus mucosa, lungs, and thyroid (22) (Fig. 1B).

Next, we investigated the expression dynamics of GBP1P1 in more detail. A time course analysis of GBP1P1 expression was performed on A549 cells infected with IAV. The results showed that GBP1P1 expression increased as early as 6 hpi, and its level continued to increase with the progression of IAV infection (Fig. 1C). Meanwhile, the upregulation of interferon beta 1 (Ifnb1) and IAV M mRNA expression was used as a confirmation of active viral infection (Fig. 1C). We then determined GBP1P1 expression in A549 cells infected with different MOIs or the different IAV strains. GBP1P1 was upregulated in an IAV dose-dependent manner, with a 70-fold increase at the highest MOI infection, as shown in Fig. 1D. In addition to the influenza A/PR/8/34 (H1N1) virus, infection with other IAV strains, including A/WSN/1933 (WSN), A/Lufang/9/93 (H3N2), and A/Jingfang/86(H1N1）isolates, also significantly increased the expression of GBP1P1 (Fig. 1E). Interestingly, GBP1P1 was expressed in different human cell lines, and its expression was dramatically upregulated after IAV infection in the A549, THP-1, and H1299 cells with a strong innate immune response to IAV invasion (Fig. 1F; Fig. S1). Together, these data demonstrate that IAV infection stimulates the expression of GBP1P1.

### Expression of GBP1P1 is strongly induced by double-strand RNA poly (I:C) and IFN-β

**Fig 2:**
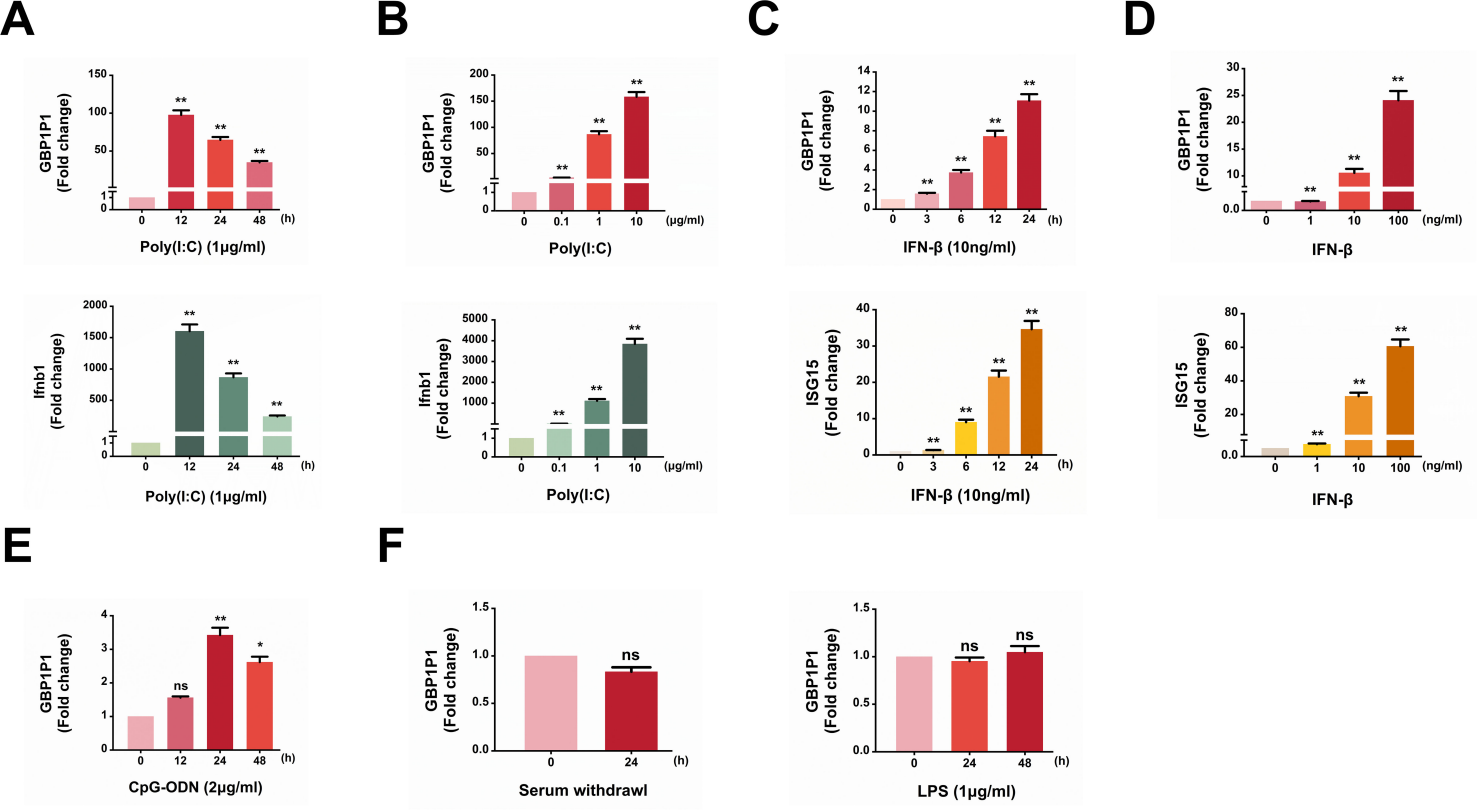
Expression of GBP1P1 pseudogene is strongly induced by double-strand RNA poly (I:C) and IFN-β. (**A**) A549 cells were transfected with poly (I:C) at a dose of 1 µg/mL, and RNA was isolated for the times indicated. (**B**) A549 cells were transfected with different concentrations of poly (I:C) for 24 h. Levels of GBP1P1 and Ifnb1 were determined by qRT-PCR (**A, B**). (**C**) A549 cells were treated with IFN-β (10 ng/mL) for the indicated hours. (**D**) A549 cells were treated with different amounts of IFN-β for 12 h. Levels of GBP1P1 and ISG15 were examined by qRT-PCR (**C, D**). (**E**) A549 cells were transfected with CpG-ODN at a dose of 2 µg/mL for the indicated times, and expression levels of GBP1P1 were measured using qRT-PCR. (**F**) A549 cells were cultured in serum-free media or incubated with LPS (1 µg/mL) for 24 h. The expression of GBP1P1 was determined by qRT-PCR. All shown qRT-PCRs are representative of three independent experiments with similar results. Results were normalized to GAPDH. Data are shown as means ± SEM. NS is not significant.*P < 0.05; **P < 0.01 vs 0 h or control.

Since IAV induced the expression of GBP1P1 immediately within a few hours after infection, we speculated that GBP1P1 expression might be induced by antiviral innate immunity. Therefore, we wondered whether IAV-activated innate immune signaling was responsible for the upregulation of GBP1P1. To test this idea, we transfected A549 cells with poly(I:C) (TLR3 agonist), a synthetic analog of double-stranded RNA (dsRNA) used experimentally in preclinical models to mimic the replication intermediates present in RNA virus-infected cells, to examine the effect of dsRNA on the induction of GBP1P1. Indeed, we observed that poly(I:C) markedly induced the expression of GBP1P1 in a time- and dose-dependent manner (Fig. 2A and B). Furthermore, we noticed that GBP1P1 could be upregulated by IFN-β in a time- and dose-dependent manner. As a positive control, the mRNA levels of the interferon-stimulated gene 15 (ISG15) also increased dramatically in the A549 cells. (Fig. 2C and D). Meanwhile, we tested the effect of the CpG-ODN (TLR9 agonist) treatment on the levels of GBP1P1. The results showed a minor increase (<fourfold) in the GBP1P1 levels in CpG-ODN-transfected cells (Fig. 2E). In addition, GBP1P1 levels were not affected by LPS (TLR4 agonist) treatment or serum deprivation (Fig. 2F). Collectively, these results suggest that an increase in GBP1P1 levels is associated with the type I IFN signaling triggered by IAV.

### IAV-induced expression of GBP1P1 involves JAK/STAT activation

**Fig 3:**
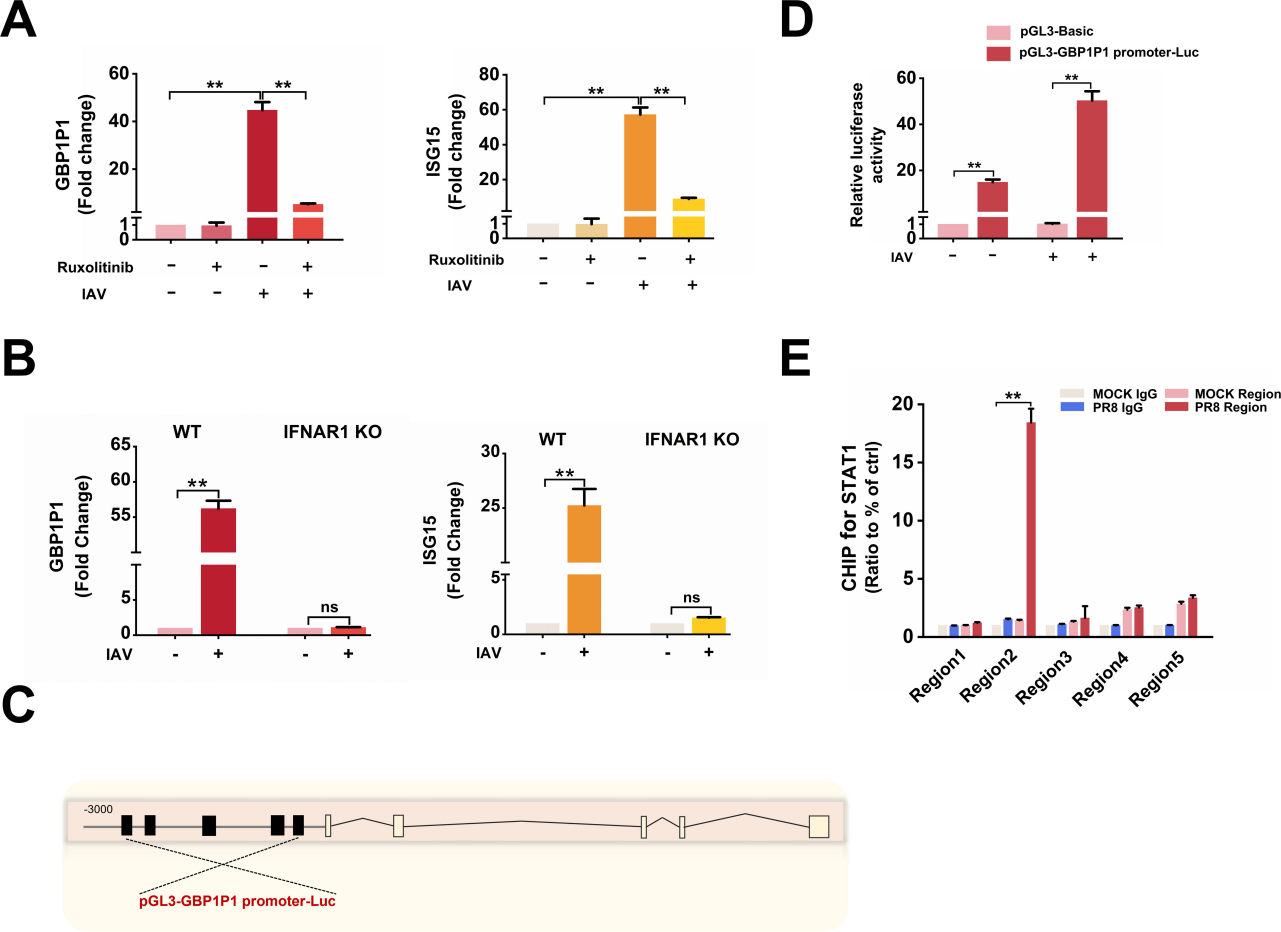
IAV-induced expression of GBP1P1 involves JAK/STAT activation. (**A**) A549 cells were treated with ruxolitinib (5 µM) or the DMSO control 30 min before IAV infection. The RNA levels of GBP1P1 in A549 cells infected or not infected with IAV for 24 h were determined by qRT-PCR. Results were normalized to GAPDH. Data are shown as means ± SEM. **P < 0.01 vs cells of noninfected control or ruxolitinib treatment. (**B**) The expression of GBP1P1 and ISG15 in WT or IFNAR1 KO A549 cells infected with IAV for 24 h was measured by qRT-PCR. Data are shown as means ± SEM. NS is not significant.*P < 0.05; **P < 0.01 vs 0 h or control. (**C**) Schematic representation of the STAT1-binding site in the human GBP1P1 gene. The black box shows the potential binding site of STAT1. The 3-kb upstream of the transcription start site of GBP1P1 was cloned and inserted into the pGL3-basic luciferase reporter construct with the putative STAT1-binding sites. (**D**) A549 cells were cotransfected with pGL3-GBP1P1 promoter-Luc reporter plasmid and pRL-CMV plasmid and then infected with IAV for 12 h. The cell lysates were harvested for the luciferase assay. After normalization with cotransfected renilla luciferase activity, relative luciferase activity was shown as fold induction of GBP1P1 promoter activity compared to A549 cells transfected with pGL-basic. Data are shown as means ± SEM. **P < 0.01 vs pGL-basic. (**E**) ChIP assays were performed in A549 cells with an anti-pSTAT1 Tyr701 antibody. Enrichment of STAT1 at the promoters of GBP1P1 is shown relative to input DNA (% input). Data represent mean and SEM of *n* = 3 biological replicates and are representative of at least two independent experiments. **P < 0.01 vs mock IgG.

Since GBP1P1 was highly expressed in A549 cells following poly(I:C) and IFN-β, we speculated that the activation of the JAK/STAT pathway might be involved in the transcription of GBP1P1 upregulated by IAV. To test this hypothesis, we performed pharmacological and genetic analyses. We exposed A549 cells to IAV in the presence of ruxolitinib, a JAK1/2 inhibitor that prevents STAT1 and STAT2 phosphorylation and suppresses the expression of ISGs. As shown in Fig. 3A, ruxolitinib blocked the upregulation of GBP1P1 induced by IAV. Consistently, GBP1P1 was not induced in IFNAR knockout (KO) A549 cells infected with IAV (Fig. 3B). The interferon-stimulated gene ISG15 was also not upregulated in IFNAR KO cells, verifying their IFN signaling deficiency (Fig. S2).

Based on ALGGEN-PROMO (https://alggen.lsi.upc.es/) and JASPER (https://jaspar.genereg.net/) database searches, putative STAT1 binding sites were identified within the potential promoter region of the GBP1P1 gene (Fig. 3C). We then cloned the potential promoter region of the GBP1P1 gene and inserted the sequence into the luciferase reporter vector. (Fig. 3D). Moreover, chromatin immunoprecipitation (ChIP) analysis revealed a significant enrichment of STAT1 in the putative promoter region-2 (Chr1:150161930–150162133) of the GBP1P1 gene locus in A549 cells after IAV infection (Fig. 3E). Collectively, these results suggest that JAK/STAT pathway is involved in the transcription of GBP1P1 induced by IAV.

### GBP1P1 represses IAV replication

**Fig 4:**
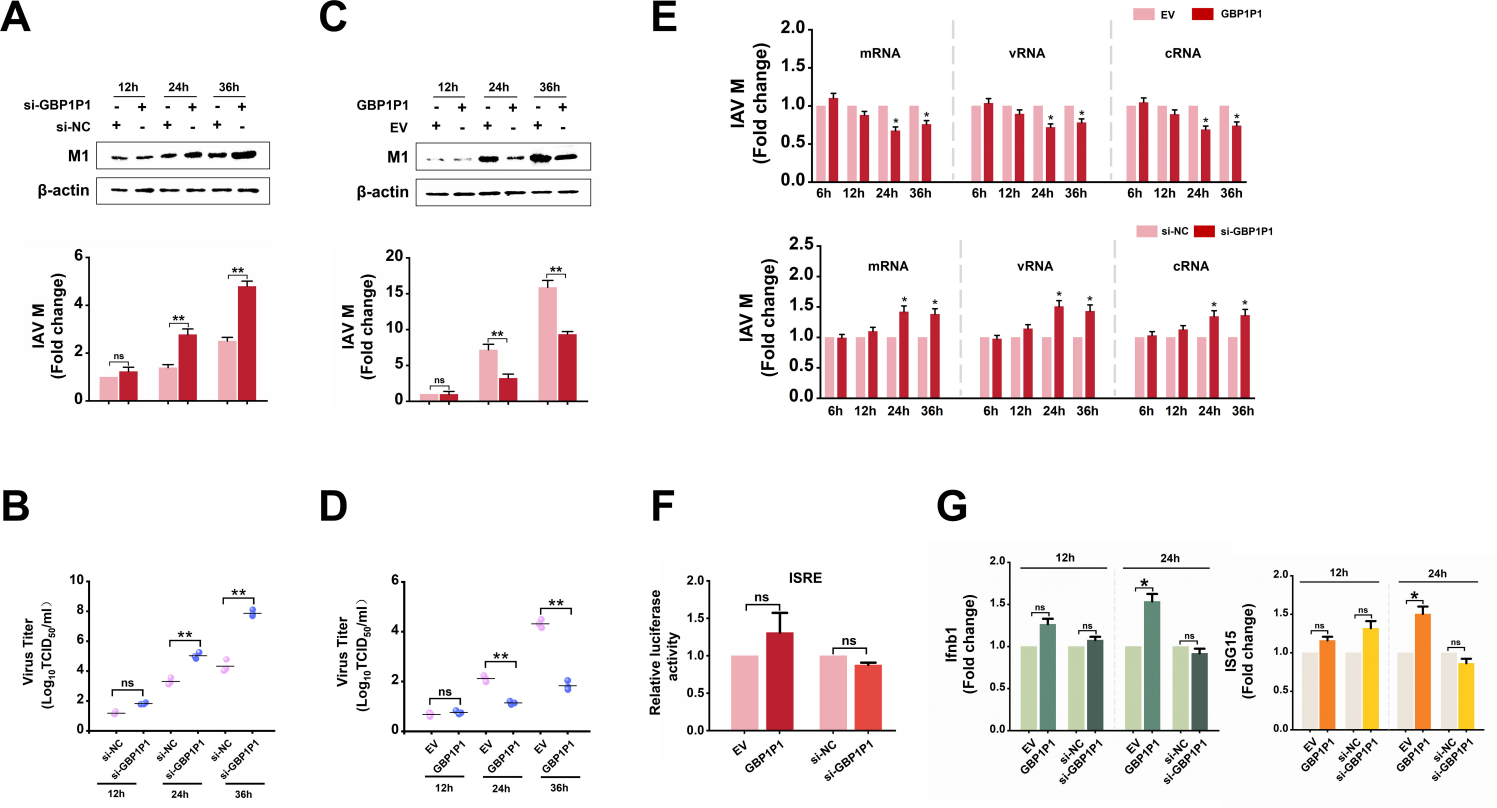
GBP1P1 represses IAV replication. (**A**) si-GBP1P1 (100 nmol) was transfected into cells in the six-well plate as indicated. A549 cells expressing si-NC were used as controls. At 12, 24, and 36 hpi, cell lysates were harvested, and levels of viral M1 proteins were analyzed using western blotting. (**B**) Culture supernatants were collected at 12, 24, and 36 hpi. Viral productions were measured using a TCID50 assay performed on MDCK cells. (**C**) pcDNA3-GBP1P1 (2 µg) was added to the transfection in the six-well plate as indicated. A549 cells expressing EV were used as controls. At 12, 24, and 36 hpi, cell lysates were harvested, and levels of viral M1 proteins were analyzed using western blotting. (**D**) Virus titers in supernatants were measured at 12, 24, and 36 hpi. (**E**) A549 cells were transfected with plasmids or siRNAs indicated. Total RNA was extracted at 6, 12, 24, and 36 hpi. qRT-PCR was used to analyze the expression of viral M mRNA, vRNA, and cRNA. (**F**) A549 cells were cotransfected with ISRE-Luc reporter plasmid, pRL-CMV plasmid, the indicated plasmids or siRNAs, and then infected with IAV for 12 h. The cell lysates were harvested for the luciferase assay. Results were normalized to renilla luciferase activity. (**G**) A549 cells were transfected with plasmids or siRNAs indicated. Relative expression levels of Ifnb1 and ISG15 mRNAs in A549 cells at 12 and 24 hpi were determined by qRT-PCR. Cells expressing EV or si-NC were used as controls. Data are shown as means ± SEM (*n* = 3; NS, not significant. *P < 0.05; **P < 0.01).

Given the high GBP1P1 expression in A549 cells infected with IAV, we further investigated the effect of the altered expression of GBP1P1 on the IAV replication. The suppressive effect of the siRNA and promoting effects of pcDNA 3-GBP1P1 on the expression of GBP1P1 was confirmed by RT-PCR and qRT-PCR (Fig. S3A and B). In a time course analysis, compared to cells transfected with negative control (NC) siRNA, A549 cells transfected with si-GBP1P1 were able to promote IAV M1 protein production much more significantly at late times (24 and 36 h) than at early times (12 h) post-infection (Fig. 4A). In addition, GBP1P1 knockdown strongly increased the virus titer as determined by the TCID50 assay (Fig. 4B). Since GBP1P1 knockdown markedly enhances IAV replication, we further detected whether ectopically expressed GBP1P1 suppresses IAV replication. To this end, A549 cells were transfected with pcDNA 3-GBP1P1 to increase the levels of GBP1P1. As shown by the results of western blotting, GBP1P1 led to a significant decrease in the IAV M1 protein also at late time points (Fig. 4C). Similarly, we also observed that overexpression of GBP1P1 significantly inhibited virus titers (Fig. 4D). In addition, we transfected A549 cells with different amounts of pcDNA 3-GBP1P1. The results showed that GBP1P1 led to a significant decrease in the IAV M1 protein and virus titers in a dose-dependent manner. In contrast, increasing amounts of si-GBP1P1 in transfections caused a gradual increase in the levels of M1 protein and virus titers (Fig. S3C-F). Similar results were obtained from GBP1P1 overexpression and knockdown in H1299 cells (Fig. S4).

Of note, disruption of GBP1P1 did not distinctly affect the synthesis of all three species of viral transcripts (mRNA, vRNA, and cRNA) in the early stage of the virus life cycle (6 and 12 hpi) as measured by strand-specific qRT-PCR. Furthermore, GBP1P1 expression has a moderate effect on viral transcription after 24 hpi (Fig. 4E; Fig. S5). These results suggest that such an inhibitory effect of GBP1P1 on IAV replication may not directly occurs at the transcription stage. In addition, the results showed that silencing or overexpression of GBP1P1 also had no effect on the luciferase activity of the IFN-stimulated response element (ISRE) and had only a slight effect on the expression of Ifnb1 and ISG15 after IAV infection when GBP1P1 was overexpressed (Fig. 4F and G). Furthermore, when GBP1P1 was silenced or overexpressed, it did not appear to have any significant impact on other ISGs, as shown in Fig. S6. These findings collectively indicate that induction of GBP1P1 may inhibit IAV replication in a manner independent of innate antiviral responses.

### Identification of DHX9 as a binding partner for GBP1P1

**Fig 5:**
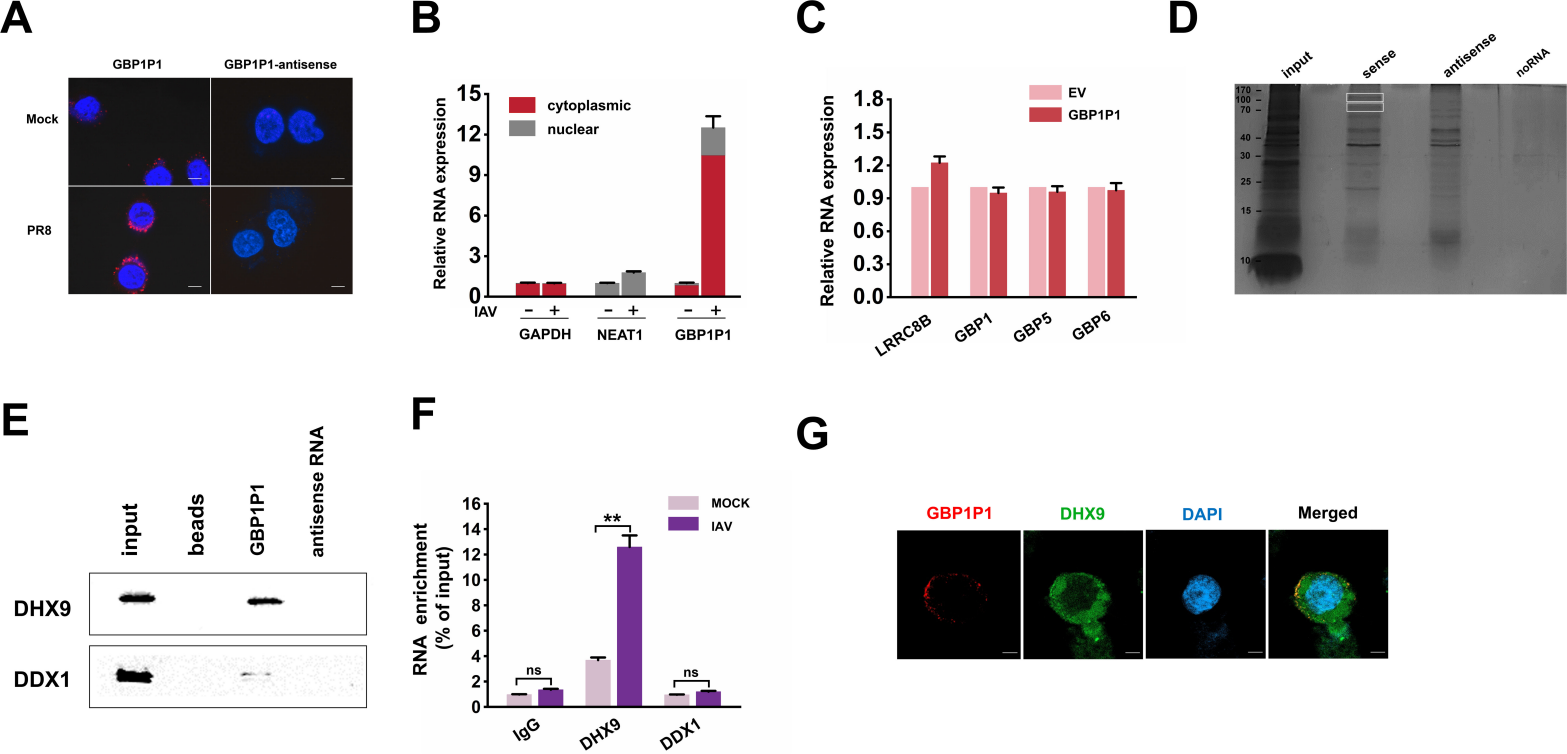
Identification of DHX9 as a binding partner for GBP1P1. (**A**) RNA FISH and confocal images showed the localization of endogenous GBP1P1 (in red) in A549 cells infected with or without IAV for 24 h. Nuclei were stained with DAPI (blue). Scale bar, 5 µm. (**B**) A549 cells were infected with or without IAV for 24 h. qRT-PCR analysis was performed to analyze the indicated RNA levels in cytoplasmic and nuclear subcellular fractionation. GAPDH mRNA and U6 RNA were used as controls for cytoplasmic and nuclear RNA, respectively. The total RNA was used as input control. Data are shown as % input (means ± SEM; *n* = 3). (**C**) A549 cells were transfected with the indicated plasmids and then infected with IAV infection for 24 h. Levels of several nearby genes of GBP1P1 were detected by qRT-PCR. Results were normalized to GAPDH. (**D**) SDS-PAGE analysis of proteins purified from *in vitro* binding assay using biotinylated GBP1P1 or antisense control RNA and A549 cytoplasmic extracts. The highlighted protein bands were subjected to mass spectrometry analysis. (**E**) RNA pull-down assay was performed to detect the association of GBP1P1 with DHX9 protein. The A549 cell lysates were incubated with *in vitro-*transcribed GBP1P1 or antisense control RNA. The pull-down products were examined by western blotting with the indicated antibodies. (**F**) RIP experiment was performed to verify that GBP1P1 co-immunoprecipitated with DHX9 protein. A549 cells were infected with IAV for 24 h. Cell lysates were immunoprecipitated with the indicated antibodies, and levels of GBP1P1 were determined by qRT-PCR. IgG was used in immunoprecipitation; the results serve as the control. (**G**) Double FISH and confocal images showed the co-localization of endogenous GBP1P1 (in red) and DHX9 (in green) in A549 cells infected with IAV for 24 h. Nuclei were stained with DAPI (blue). Scale bar, 5 µm. All shown qRT-PCRs are representative from at least two independent experiments with similar results. **P < 0.01 vs mock-treated cells.

To elucidate the mechanism of action of GBP1P1, we first determined the subcellular location and half-life of GBP1P1 in A549 cells. RNA FISH and qRT-PCR of cell fractions showed that GBP1P1 is located in the cytoplasm (Fig. 5A and B). We also detected whether GBP1P1 acts *in cis*, affecting nearby gene expression. qRT-PCR quantification analysis showed that overexpression of GBP1P1 did not dramatically regulate the expression of nearby genes, including LRRC8B, GBP1, GBP5, and GBP6 (Fig. 5C). These data suggest that GBP1P1 may interact with molecules or proteins in the cytoplasm.

We next sought to identify the protein partners of GBP1P1. We performed RNA–protein-binding assays by incubating *in vitro*-transcribed biotinylated GBP1P1 or its antisense control RNA with cytoplasmic extracts from A549 cells. RNA–protein complexes were captured using streptavidin magnetic beads and resolved by SDS-PAGE. Protein bands that were specifically enriched in the GBP1P1 pull-down were subjected to mass spectrometry for identification (Fig. 5D). This approach identified 33 RNA-binding proteins enriched in GBP1P1 pull-downs compared to antisense controls (Table S3). Considering that GBP1P1 did not noticeably affect the production of three different RNA species of IAV, several multifunctional proteins caught our attention. The ability of DHX9 [also known as nuclear DNA Helicase II (NDH II) and RNA Helicase A (RHA)] to bind GBP1P1 was confirmed using western blotting (Fig. 5E).

As the RNA pull-down assay was performed in cell lysate and not in a native cellular environment, the interaction between GBP1P1 and DHX9 could have been an artifact of the assay conditions. To address this issue, we performed RIP assays, in which antibodies against DHX9 were incubated with A549 lysates to pull down the endogenous proteins, followed by qRT-PCR analysis to examine the levels of GBP1P1 retrieved by each immunoprecipitation. The results showed that GBP1P1 could be enriched in DHX9 precipitates, providing further evidence that GBP1P1 interacts with DHX9 protein (Fig. 5F). The RNA-binding protein DDX1 was used as a control. The interaction between GBP1P1 and DHX9 was also confirmed by double FISH. Notably, obvious co-localization of GBP1P1 and DHX9 was found in the cytoplasm of A549 cells infected with IAV (Fig. 5G). These results indicate that GBP1P1 specifically interacts with DHX9.

### GBP1P1 suppresses IAV replication via interaction with DHX9

**Fig 6:**
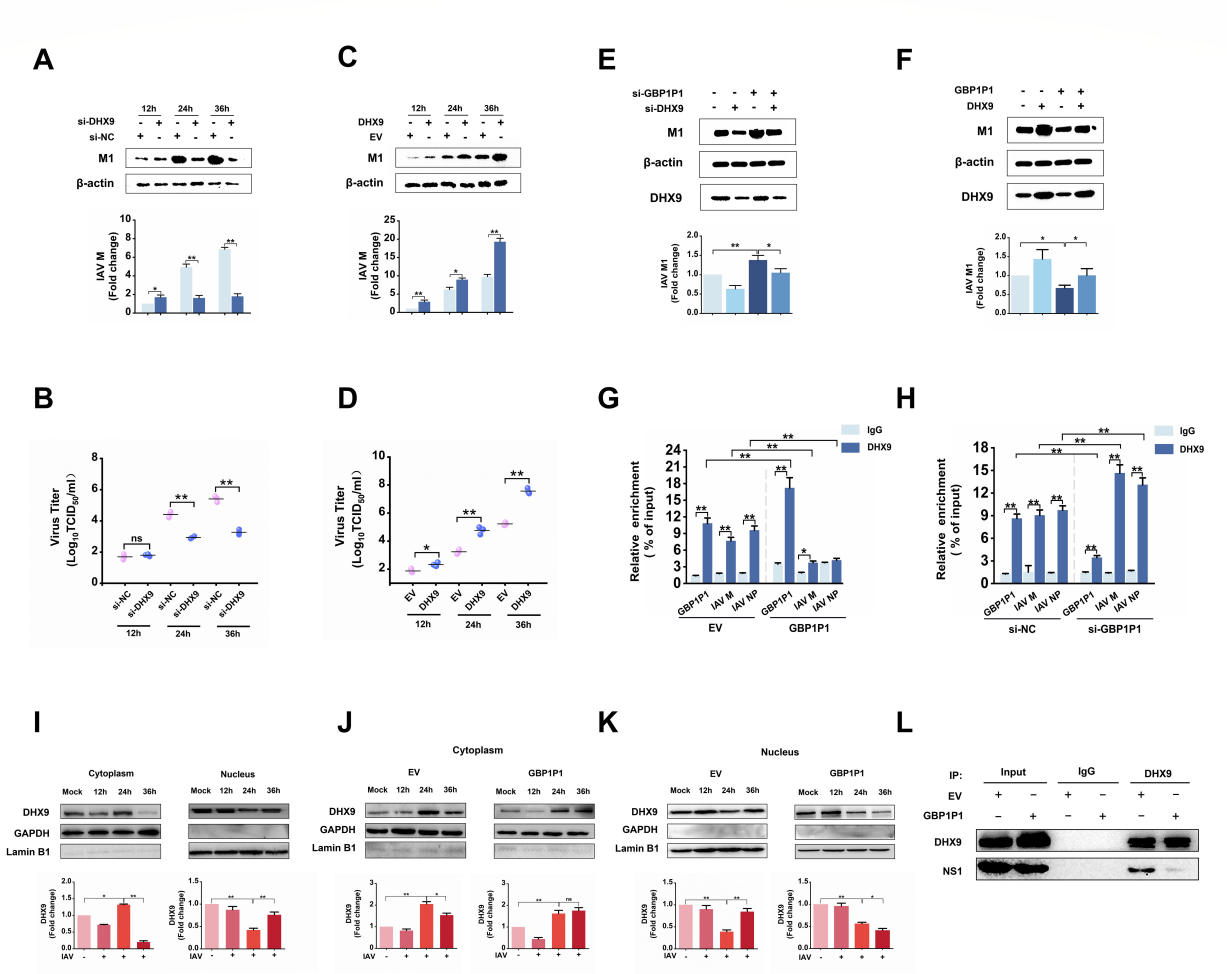
GBP1P1 suppresses IAV replication via interaction with DHX9. A549 cells were transfected with the indicated siRNAs (**A, B**) or the indicated plasmids (**C, D**) and then infected with IAV. At 12, 24, and 36 hpi, cell lysates and culture supernatants were collected. Levels of viral M1 proteins were analyzed using western blotting (**A, C**). β-actin was used as a loading control. Viral productions were measured by a TCID50 assay performed on MDCK cells (**B, D**). A549 cells were cotransfected with the indicated siRNA (**E**) or the indicated plasmids (**F**) for 24 h and then infected with IAV for 36 h. Levels of viral M1 protein in cell lysates were detected using western blotting. A549 cells were transfected with GBP1P1-expressing vector (**G**) or GBP1P1 siRNA (**H**) for 24 h and then infected with IAV for 24 h. RIP assays were performed using a DHX9-specific antibody. qRT-PCR was used to examine GBP1P1, viral M1 mRNA, and NP mRNA binding to DHX9. (**I**) Examination of DHX9 location in A549 cells infected with IAV for 12, 24, and 36 h. (**J, K**) A549 cells were transfected with pcDNA3-GBP1P1 or EV and then infected with IAV for the indicated times. A cytoplasmic and nuclear fractionation kit was used to obtain the cytosolic and nuclear proteins. The subcellular localization of DHX9 was analyzed using Western blotting. GAPDH and LaminB1 were considered as cytosolic and nuclear loading controls, respectively. (**L**) A549 cells were transfected with the plasmids indicated and then infected with IAV. Cell lysates were prepared at 24 hpi, precleared by IgG, and incubated with either anti-DHX9 antibody or IgG. Immunoprecipitated proteins were subjected to western blotting with DHX9 and NS1 antibodies. Ten percent of the input was loaded as a control. Data represent mean and SEM of *n* = 3 biological replicates. *P < 0.05, **P < 0.01. NS, not significant.

Because GBP1P1 suppresses IAV replication and binds to DHX9, we determined whether DHX9 plays a role in IAV replication. We found that the knockdown of DHX9 significantly decreased IAV replication, as determined by western blotting and TCID50 assay (Fig. 6A and B). Complementarily, overexpression of DHX9 resulted in an increased infection burden in A549 cells (Fig. 6C and D). Similar results were obtained from DHX9 overexpression and knockdown in H1299 cells (Fig. S7). We then checked whether or not GBP1P1 functions through DHX9. As shown in Fig. 6E, silencing DHX9 in A549 cells partially rescued the cells from the effects of GBP1P1 siRNA on IAV replication, as indicated by the M1 expression levels. Furthermore, supplementing the cells with recombinant DHX9 rescued the effect of GBP1P1 overexpression on viral replication (Fig. 6F). Based on these data, we propose that GBP1P1 suppresses the replication of IAV, at least in part, through its interaction with DHX9.

**Fig 7:**
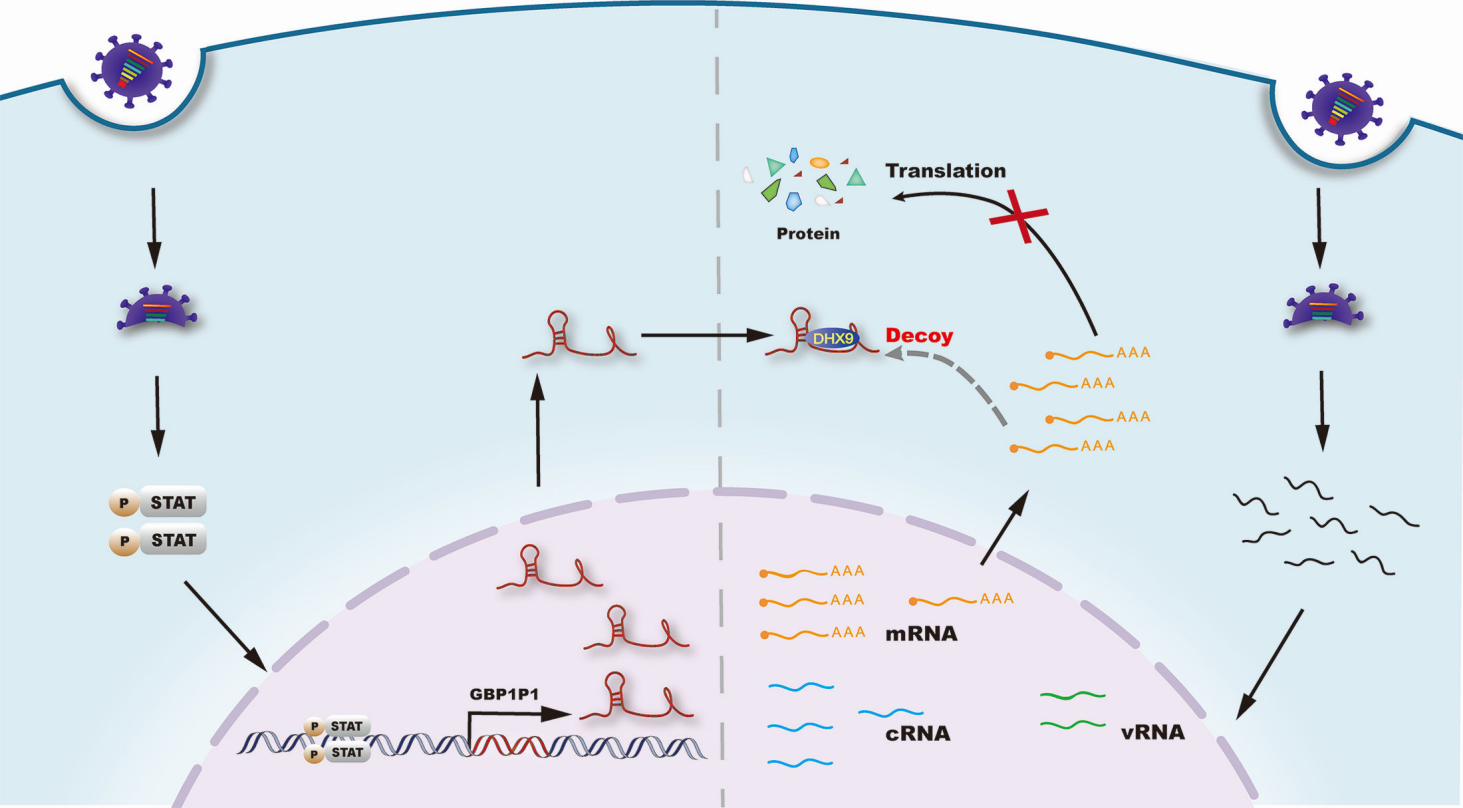
Proposed molecular mechanism for the function of GBP1P1 to suppress IAV replication. Upon IAV infection, GBP1P1, a host pseudogene-derived lncRNA, is highly upregulated through activation of the JAK–STAT signaling pathway. GBP1P1 functions as a decoy to titrate DHX9 way from viral mRNA in the cytoplasm and further attenuate IAV replication.

DHX9 is a well-studied RNA helicase, and one of its known functions is to participate in the regulation of gene expression at the translational level (23–28). We postulate that GBP1P1 may act as a sponge to trap DHX9. To test this model, we conducted the overexpression and knockdown of GBP1P1 in A549 cells, respectively, and then performed RIP for DHX9. As shown in Fig. 6G, the overexpression of GBP1P1 resulted in a decrease in M1 mRNA binding to DHX9. Complementarily, the reduction in GBP1P1 expression led to an increased enrichment of M1 mRNA by DHX9 IP (Fig. 6H). Moreover, we observed a dynamic nucleocytoplasmic distribution of DHX9 in response to IAV infection in A549 cells, characterized by its translocation from the nucleus to the cytoplasm at 24 hpi (Fig. 6I). Overexpression of GBP1P1 significantly altered the distribution pattern of DHX9 (Fig. 6J and K), further supporting that GBP1P1 interacts with DHX9 in the cytoplasm. Additionally, we detected the protein levels of DHX9 in GBP1P1-silenced or overexpressed cells, indicating that GBP1P1 also has no significant effect on DHX9 expression (Fig. S8). A previous study has demonstrated that DHX9 interacts with viral nonstructural protein 1 (NS1) and enhances viral replication and transcription (29). Using the Co-IP experiment, we also investigated whether GBP1P1 can interfere with the DHX9–NS1 interaction. The results proved that GBP1P1 overexpression significantly disrupted the interaction between DHX9 and viral NS1 protein (Fig. 6L). These data support that GBP1P1 functions through the competition model (Fig. 7).

## DISCUSSION

IAV is a widespread zoonotic pathogen that poses a threat to the health of both humans and animals. IAV infection in humans can cause illness ranging from mild upper respiratory tract infection to severe pneumonia and even death (30, 31). Over the past few decades, the main focus of research has been on the role of protein-coding genes in viral pathogenesis. However, recent advances in high-throughput transcriptome sequencing have revealed the differential expression of pseudogenes in multiple diseases, including viral infections (32–39). Viral infection results in the downregulation of cellular 5S ribosomal RNA pseudogene 141 (RNA5SP141)-interacting proteins, thereby allowing RNA5SP141 to bind RIG-I and induce antiviral immune responses. Silencing of RNA5SP141 leads to a strong reduction in the transcription of IFNB1, IFIT1, and ISG15 and in the secretion of IFN-β and CCL5 in response to IAV (38). The interferon-induced transmembrane protein 4 pseudogene (IFITM4P), as a sponge of miR-24–3p, can promote the stability of IFITM1/2/3 mRNAs and inhibit IAV replication (34). In exosomes from IAV-infected MDCK cells, 856 RNA transcripts were markedly differentially expressed including 33 pseudogene transcripts. The majority of the 31 upregulated pseudogenes originated from genes involved in translation and gene expression (32). Here, we report that GBP1P1, a pseudogene-derived lncRNA, is expressed in various human cells, but is significantly induced upon IAV infection. Remarkably, we have revealed that the altered expression of GBP1P1 has a profound effect on IAV replication by decoying DHX9, an RNA/DNA helicase associated with multiple cellular processes such as transcription and translation regulation (23, 25, 40). These findings provide evidence that GBP1P1 functions as a sponge to trap DHX9, which modulates viral transcription and translation.

The results of a series of experiments demonstrate that the expression of GBP1P1 is regulated by an IFN-induced JAK/STAT signaling pathway, which is in accordance with a classical ISG induction. These findings led us to investigate whether GBP1P1 acts as a regulator of the JAK/STAT pathway or the expression of other GBPs. However, altering the expression of GBP1P1 did not have a significant impact on the mRNA levels of other GBPs or on the activation of the JAK/STAT signaling, suggesting that other mechanisms may be involved in the inhibition of IAV replication by GBP1P1. Meanwhile, GBP1P1 loss- and gain-of-function experiments suggest that GBP1P1 may strongly affect viral gene expression and replication, predominantly at the level of mRNA translation. In addition, GBP1P1 overexpression had only a slight effect on the expression of Ifnb1 and ISG15. Nevertheless, this increase was significantly lower in comparison to the impact observed on ISGs by the lncRNAs, which primarily function by regulating the antiviral innate immune (41, 42). Accordingly, we speculate that the induction of GBP1P1 may inhibit IAV replication independent of the innate antiviral response.

LncRNAs typically interact with proteins to exert their functions. Using RNA pull-down assay, mass spectrometry, and RIP assay, we found that GBP1P1 interacts with DHX9. DHX9 normally localizes in the nucleoplasm and is a multifunctional ATP-dependent nucleic acid helicase. Through interacting with a variety of nuclear and cytoplasmic protein and nucleic acid partners, DHX9 is involved in diversified cellular processes including DNA replication, DNA repair, transcriptional regulation, post-transcriptional RNA modification, or translational regulation (23, 25, 40). Existing evidence indicates that DHX9 can affect different steps along the RNA virus life cycle, from transcription to infectivity of progeny virions, and its involvement in supporting both viral genome replication and translation has been demonstrated in the infection of several viruses, including IAV, Chikungunya virus (CHIKV), classical swine fever virus (CSFV), HCV, foot and mouth disease virus (FMDV), and HIV-1 (28, 29, 43–47). However, several studies in recent years have shown that DHX9 can also inhibit viruses, such as Epstein–Barr virus (EBV) and rotavirus, either by serving as the viral nucleic acid sensor or by regulating downstream signaling events (48–50). This suggests that DHX9 might play multifaceted regulatory roles in the replication of various viruses.

In this study, we observed that the subcellular localization of GBP1P1 and the co-localization of GBP1P1 and DHX9 are mostly cytoplasmic in A549 cells after IAV infection. We accordingly postulate that GBP1P1, as a cytoplasmic lncRNA, may serve as a sponge to trap DXH9, implying that GBP1P1 might restrict the functions of DHX9 in the cytoplasm, such as translational regulation. Subsequently, GBP1P1 was found to compete with viral mRNA for DHX9 binding. In addition, GBP1P1 can also disrupt the interaction between DHX9 and viral NS1 protein. The interference of GBP1P1 accumulation after IAV infection on the production of viral proteins and the DHX9–NS1 interaction subsequently could impair viral transcription, which may partially explain why the inhibition of viral transcription by GBP1P1 expression does not occur in the early stage of IAV replication. This working model is reminiscent of several other lncRNAs that have a similar mechanism of action. LncRNA PIRAT (PU.1-induced regulator of alarmin transcription), acting as a nuclear decoy RNA, prevents PU.1 from binding to alarmin promoters and promotes its binding to pseudogenes in naïve monocytes, which plays an important role in regulating antiviral responses to severe acute respiratory syndrome coronavirus 2 in humans (51). IFITM4P as a ceRNA inhibits the replication of IAV via the IFITM4P-miR-24–3p-IFITM1/2/3 regulatory network (34). The cytoplasmic lnc-Lsm3b, as an inducible “self” lncRNA, acts as a molecular decoy that actively saturates RIG-I-binding sites to restrict the duration of the viral RNA-induced innate immune response and maintain immune homeostasis (52). Doublesex1-alpha-promoter-associated-long noncoding-RNA (*DAPALR*) can bind and sequester the Shep (Alan Shepard) protein away from the *Dsx1* mRNA, thereby activating *Dsx1* translation (53). Thus, the decoy model may be an effective mechanism that has been adapted by many lncRNAs to modulate their targets.

The extent to which lncRNAs function through this mechanism is still in question due to the fact that the copy number of most proteins is between 10,000 and 80,000,000 per cell, which is remarkably higher than that of lncRNAs with the expression of 0.3–1,000 molecules per cell (54). However, several mechanisms have been explored by lncRNAs to overcome their low abundance compared to their interacting proteins, such as cellular local concentration and rapid induction of expression in response to stimuli. In our decoy model, we estimated the relative quantitative relationship between GBP1P1 and DHX9 using Ct values. Although the abundance of the GBP1P1 is approximately 1/50 that of DHX9 mRNA after IAV infection, the translated DHX9 protein is predominantly a nuclear protein and is able to translocate to the cytoplasm to exert some of its translational regulatory functions, implying that an appropriate amount of GBP1P1 in the cytoplasm can at least partially sequester DXH9 away from viral mRNA to restraint IAV replication. It is important to note, however, that this decoy model does not provide a complete picture of how GBP1P1 functions. GBP1P1 is likely to have interactions with multiple proteins or miRNAs. This deserves further investigation in the future.

In summary, we demonstrate that GBP1P1 is strongly induced by IAV in a JAK/STAT-dependent manner. Furthermore, we reveal that GBP1P1 can compete with viral mRNAs for binding to DHX9 protein and thus attenuate viral translation and production. As a novel antiviral host factor, GBP1P1 may also be more broadly implicated in other viral infections. These findings advance our understanding of the physiological role of lncRNAs in viral infection.

## Data Availability

All data are available within the article and supplemental material.

## SUPPLEMENTAL MATERIAL

Supplemental material jvi.00738-24-s0001.doc Figures S1 to S8; Tables S1 to S3.

ASM does not own the copyrights to Supplemental Material that may be linked to, or accessed through, an article. The authors have granted ASM a non-exclusive, world-wide license to publish the Supplemental Material files. Please contact the corresponding author directly for reuse.
