# Supplementary material for: The pseudogene GBP1P1 suppresses influenza A virus replication by acting as a protein decoy for DHX9
Source: J Virol. 2024 Jun 28;98(7):e00738-24. doi: 10.1128/jvi.00738-24 (PMC11264600; doi:10.1128/jvi.00738-24)
Supplement: Supplemental material — Figures S1 to S8; Tables S1 to S3. [file jvi.00738-24-s0001.doc]

**Supporting Information for**

**The pseudogene GBP1P1 suppresses Influenza A Virus replication by acting as a protein decoy for DHX9**

Xiaohang Yu^1#^, Jiaxin Tian^2#^, Yihe Wang^1^, Ning Su^1^, Jinna Luo^1^, Ming Duan^1*^, Ning Shi^1*^

^1^ State Key Laboratory for Diagnosis and Treatment of Severe Zoonotic Infectious Diseases, Key Laboratory for Zoonosis Research of the Ministry of Education, Institute of Zoonosis, and College of Veterinary Medicine, Jilin University, Changchun 130062, Jilin Province, China.

^2^ School of Life Science and Technology, Changchun University of Science and Technology, Changchun, 130031, Jilin Province, China.

^#^ Xiaohang Yu and Jiaxin Tian contributed equally to this work and considered as co-first authors. Author order was determined by the duration worked on this project.

^*^ Address correspondence to Ming Duan, duan_ming@jlu.edu.cn, or Ning Shi,

shining17@jlu.edu.cn.

The authors declare no conflict of interest.


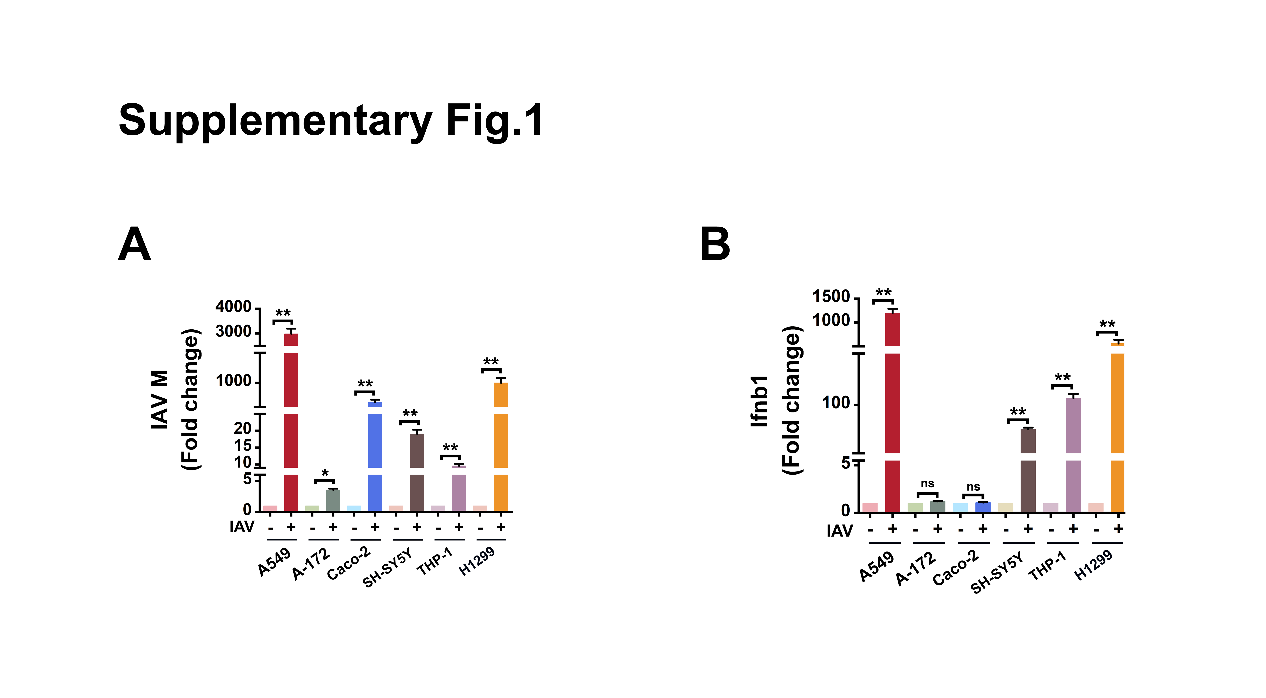


**Fig S1.** **The expression levels of IAV M and Ifnb1 across different cell lines infected with IAV.**

The expression of IAV M (A) and Ifnb1 (B) mRNA in indicated human cell lines infected with/without PR8 for 24 hours was examined by qRT-PCR. All Shown qRT-PCRs are representative from three independent experiments with similar results. Results were normalized to GAPDH. Data are shown as means ± SEM. NS, not significant.*p < 0.05; **p < 0.01 vs. mock-treated cells.


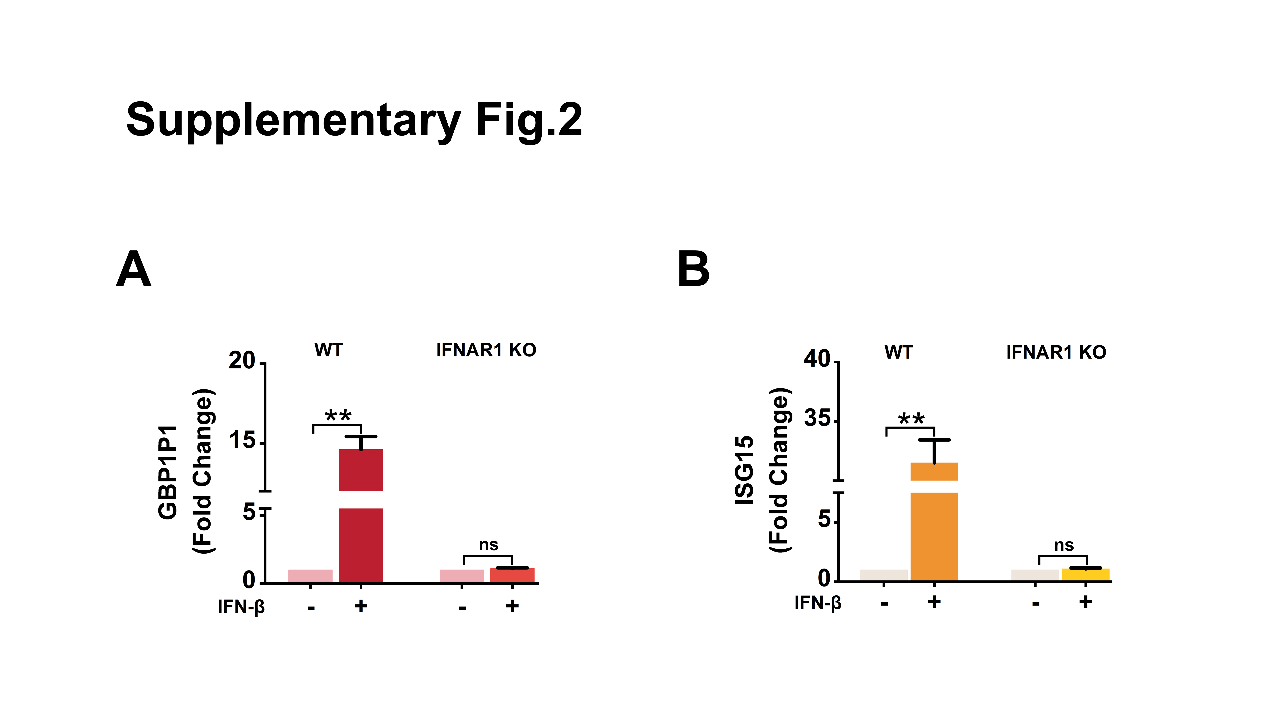


**Fig S2.** **The expression levels of GBP1P1 and ISG15 in IFNAR KO cells after IFN-β treatment.**

A549 WT or A549 IFNAR1^-/-^ cells were stimulated by IFN-β. qRT-PCR was used to analyze the expression of GBP1P1 and ISG15. Data represent mean ± SEM of n=3 biological replicates. NS, not significant.*p < 0.05, **p < 0.01. control.

**
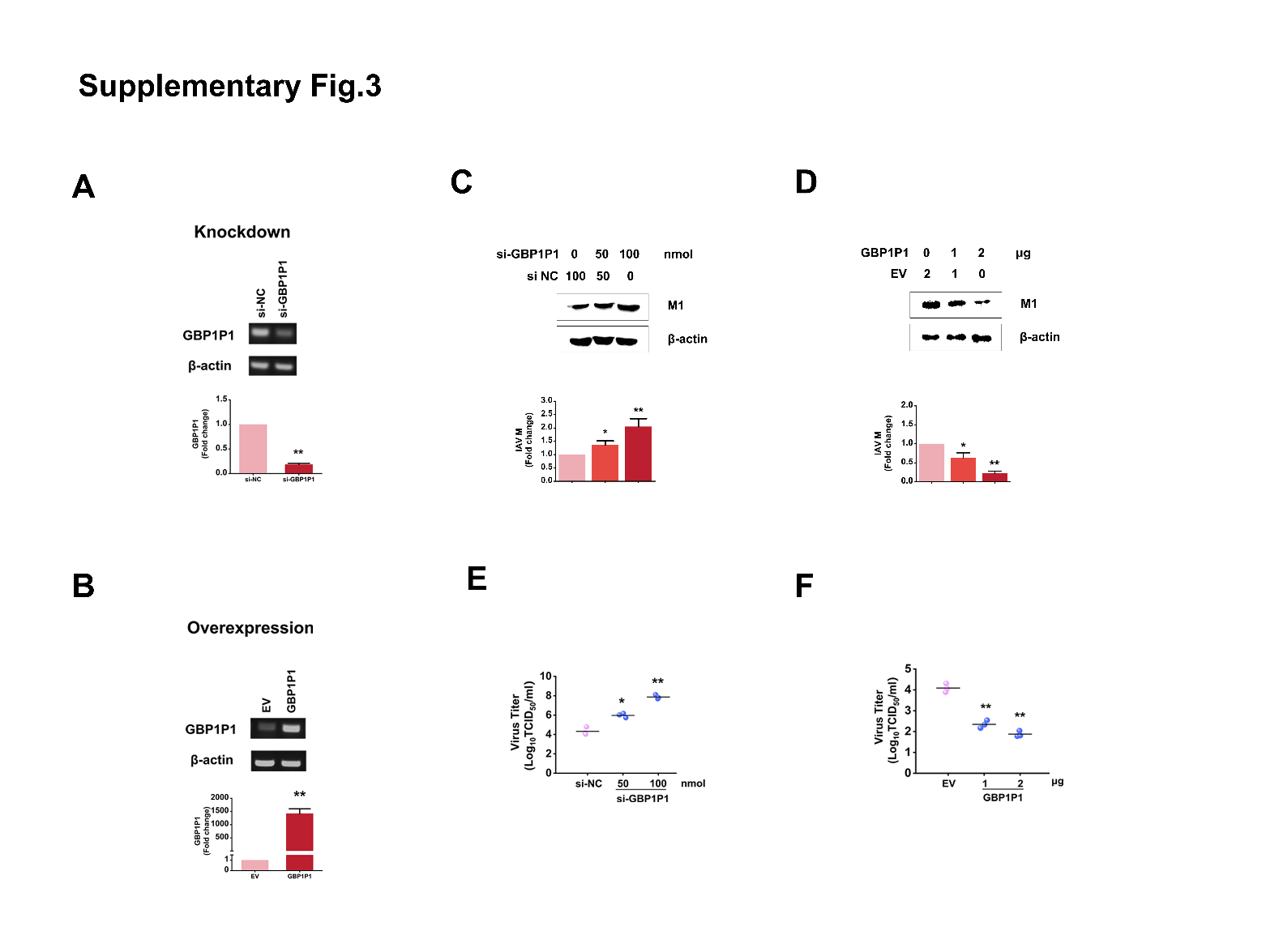
**

**Fig S3. IAV replication in A549 cells transfected with different amounts of pcDNA3-GBP1P1 or si-GBP1P1.**

The indicated siRNAs (A) or plasmids (B) were transfected into A549 cells. At 24 hpi, levels of GBP1P1 were analyzed by RT-PCR (up) and qRT-PCR (down). Results were normalized to GAPDH. Data are shown as means ± SEM. **p < 0.01 vs. control. (C) Increasing amounts of si-GBP1P1 were transfected into A549 cells in the 6-well plate as indicated. NC-siRNA was added to bring the total siRNAs to 100 nmol. At 36 hpi, cell lysates were harvested and levels of viral M1 proteins were analyzed by Western blotting. (D) Increasing amounts of pcDNA3-GBP1P1 were added to the transfection in the 6-well plate as indicated. EV was added to bring the total plasmids to 2μg. At 12, 24, 36 hpi, cell lysates were harvested and levels of viral M1 proteins were analyzed by Western blotting. (E) Culture supernatants were collected at 36 hpi. Viral productions were measured by a TCID_50_ assay performed on MDCK cells. (F) Virus titers in supernatants were measured at 36 hpi. Viral productions were measured by a TCID_50_ assay performed on MDCK cells. Data represent mean ± SEM of n=3 biological replicates. *p < 0.05, **p < 0.01 vs. control.

**
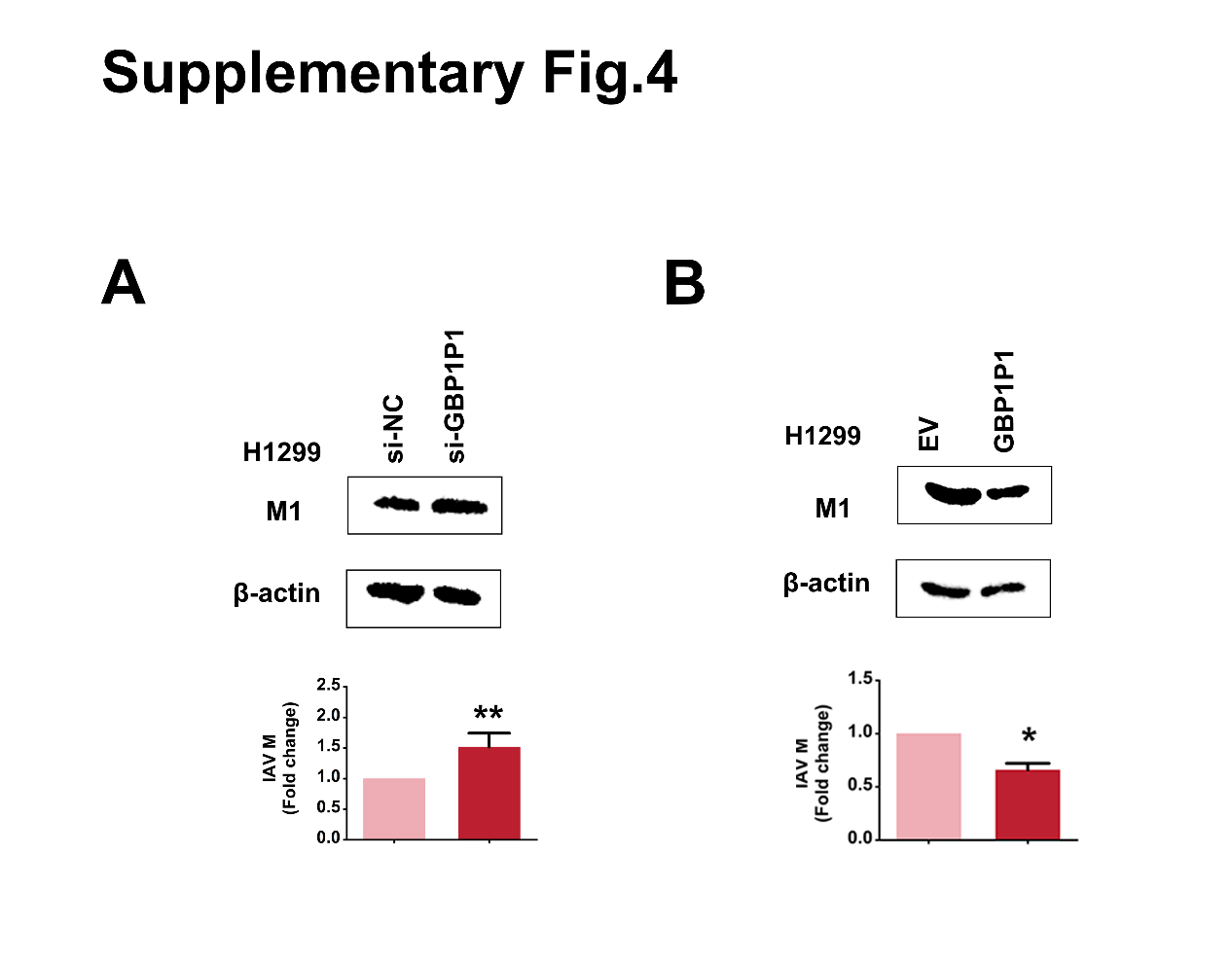
**

**Fig S4. Protein levels of IAV M1 in H1299 cells with GBP1P1 silenced or overexpressed.**

The indicated siRNAs (A) or plasmids (B) were transfected into H1299 cells. At 36 hpi, levels of M1 were analyzed by Western blotting. Data are shown as means ± SEM. *p<0.05; **p < 0.01 vs. control.


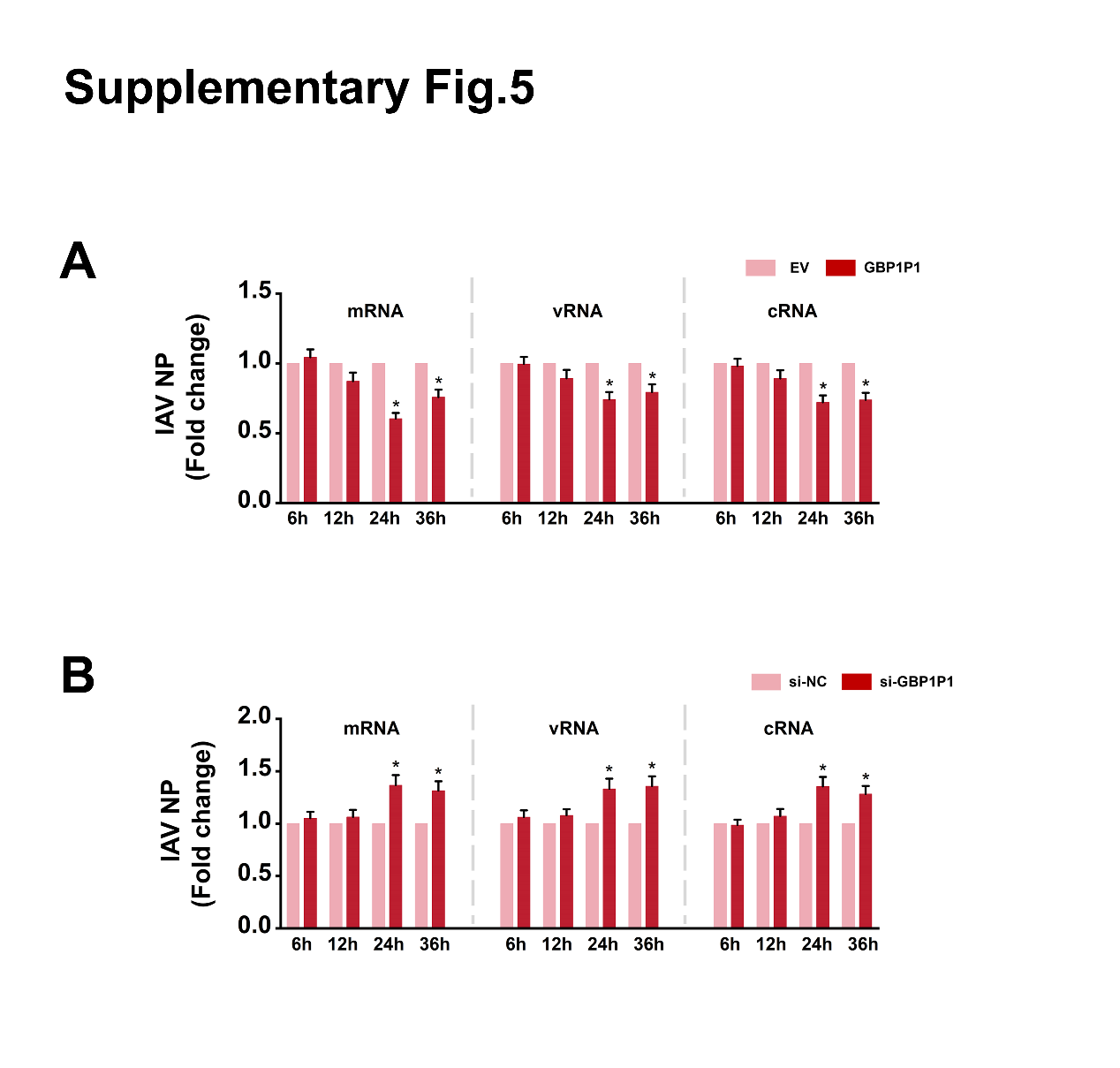


**Fig S5. Effects of GBP1P1 on viral NP gene transcription.**

The indicated plasmids (A) or siRNAs (B) were transfected into A549 cells. Total RNA was extracted at 6, 12, 24 and 36 hpi. qRT-PCR was used to analyze the levels of viral NP mRNA, vRNA, and cRNA. Data are shown as means ± SEM. *p<0.05 vs. EV or si-NC.


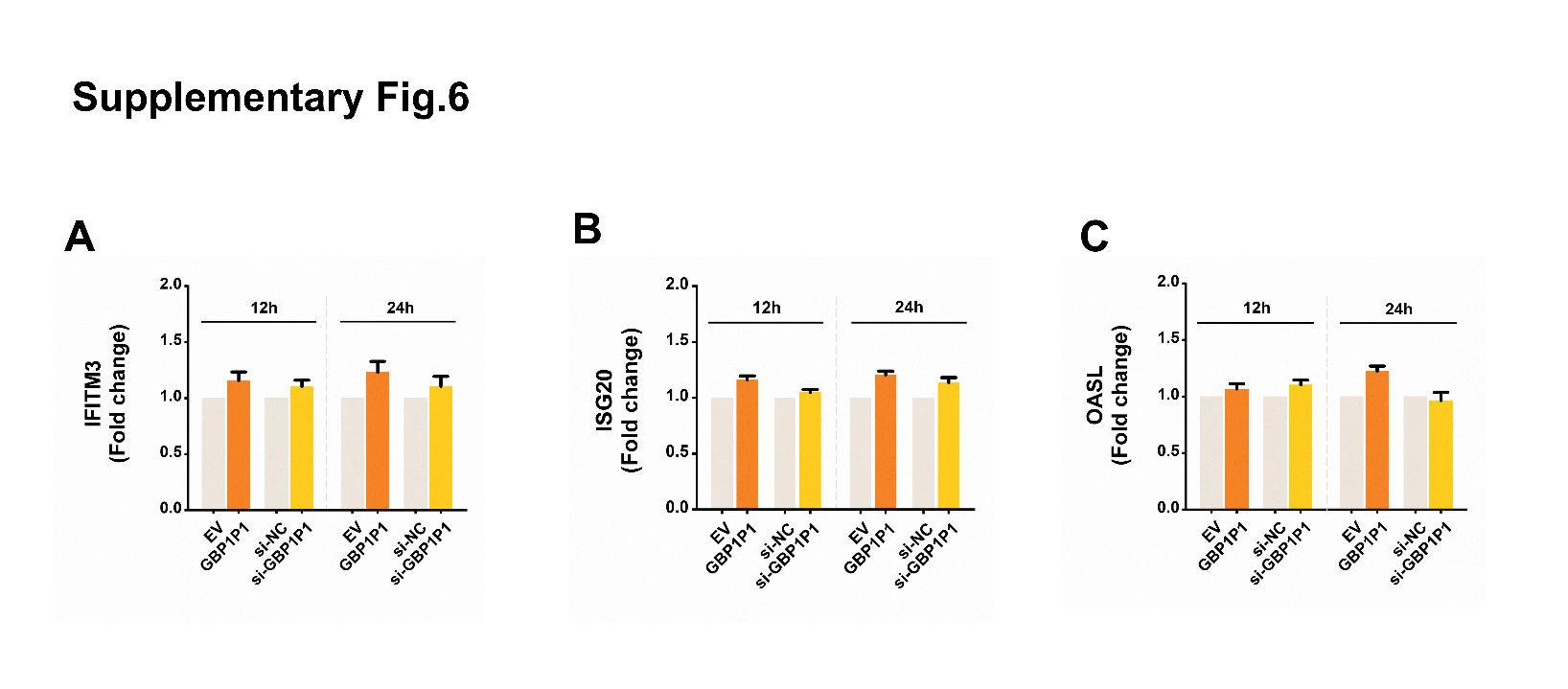


**Fig S6. Effects of GBP1P1 on IFITM3, ISG20 and OASL gene transcription.**

A549 cells were transfected with plasmids or siRNAs indicated. Relative expression levels of IFITM3 (A), ISG20 (B) and OASL (C) mRNAs in A549 cells at 12 and 24 hpi were determined by qRT-PCR. Cells expressing EV or NC-siRNA were used as controls. Data are shown as means ± SEM.


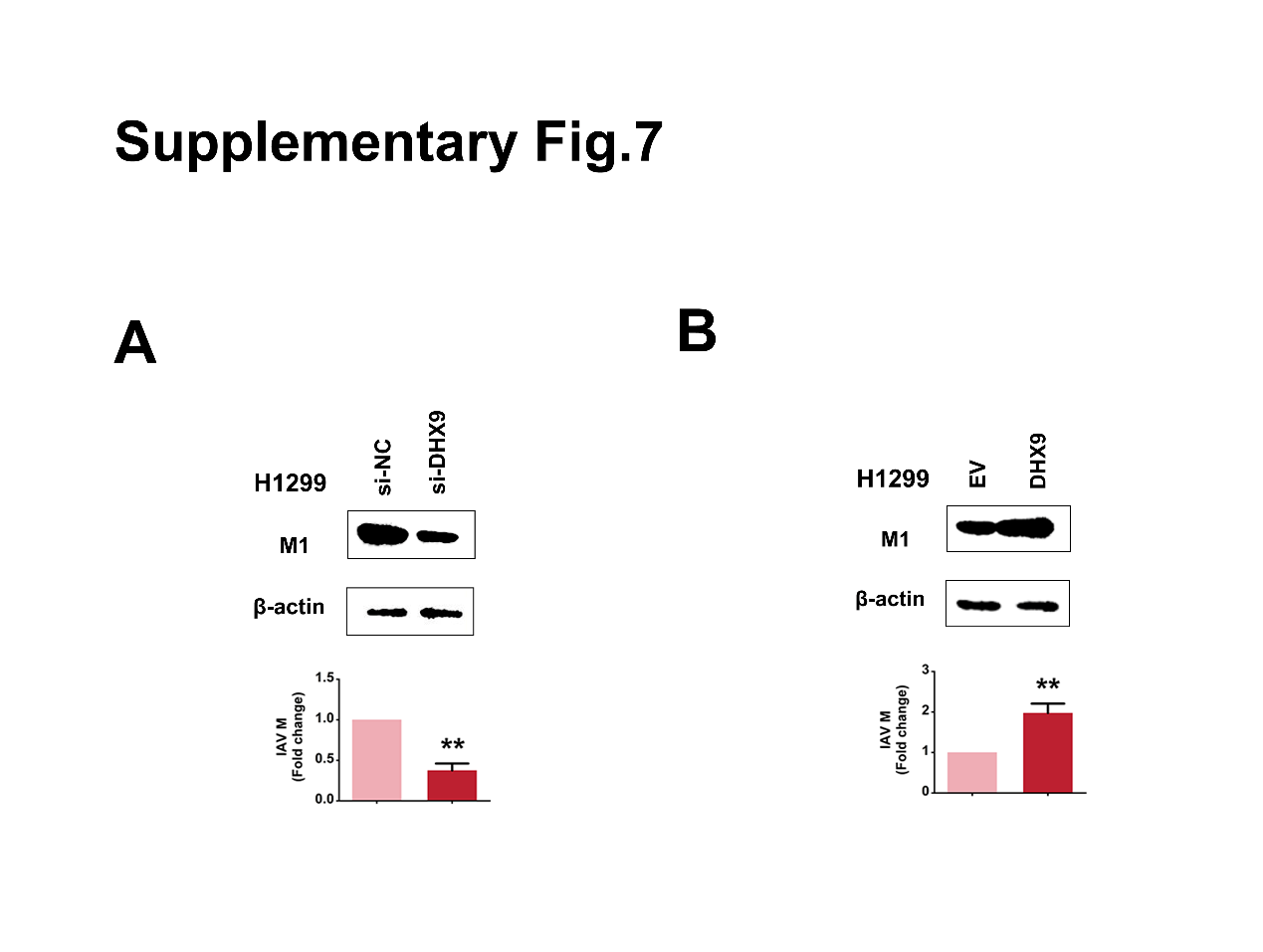


**Fig S7. Protein levels of IAV M1 in H1299 cells with DHX9 overexpressed or silenced.**

The indicated siRNAs (A) or plasmids (B) were transfected into H1299 cells cells. At 24 hpi, levels of M1 were analyzed by Western blotting. Data represent mean and SEM of n=3 biological replicates. *p < 0.05, **p < 0.01. vs. EV or si-NC.


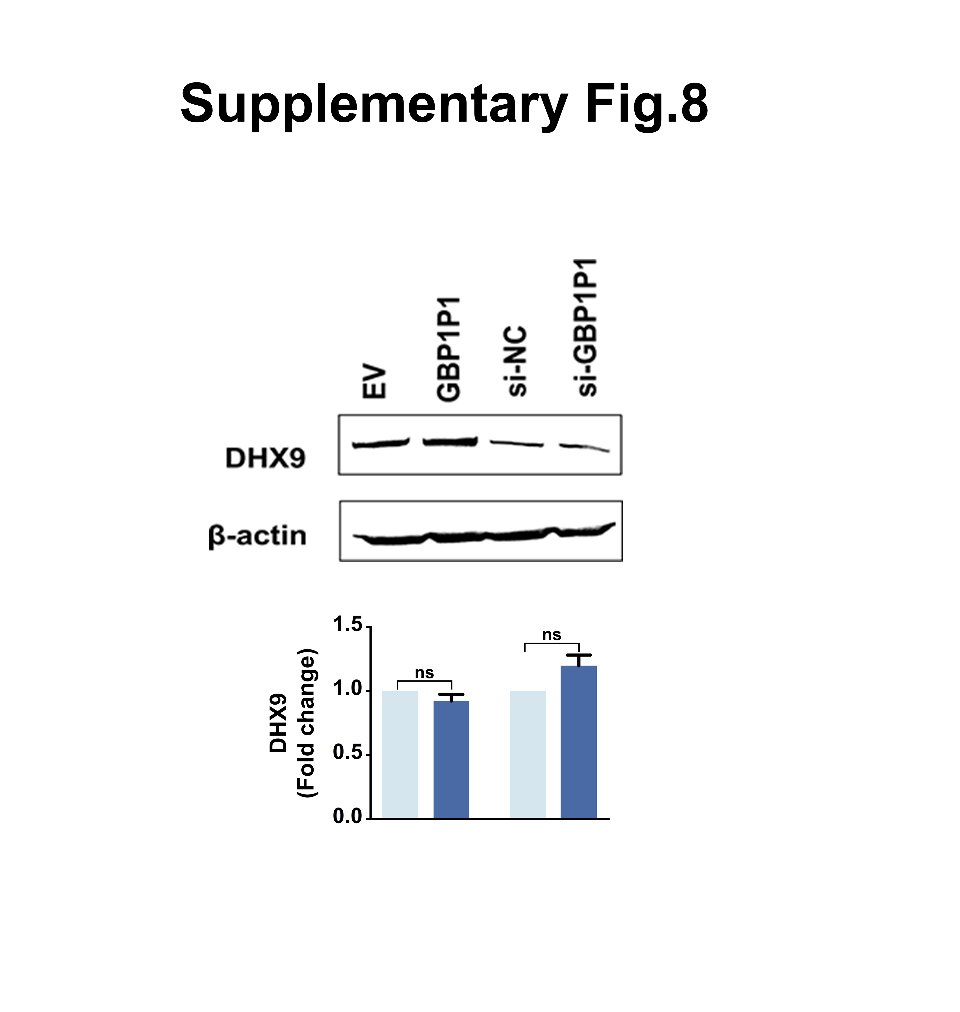


**Fig S8.** **Protein levels of DHX9 in A549 cells with GBP1P1 overexpressed or silenced.**

A549 cells were transfected with the indicated plasmids or siRNA. At 36 hours post-transfection, the cell lysates were collected for western blot analysis. β-actin was used as a loading control.

**Table S1. Primers used for quantitative real time-PCR and other assays in this study.**

| **Gene Symbol** | **Forward primer (5’→3’)** | **Reverse primer (5’→3’)** |
| --- | --- | --- |
| Ifnb1 | AGTAGGCGACACTGTTCGTG | CAATGACCCCTTCATTGACC |
| GAPDH | TTGATTTTGGAGGGATCTCG | ACCTTCTACAATGAGCTGCG |
| β-Actin | CCTGGATAGCAACGTACATGG | GACCAATCCTGTCACCTCTGA |
| IAV-M | GTATATGAGGCCCATRCAACTG | CAGCCATGGGCTGGGAC |
| ISG15 | CTTCAGCTCTGACACCGACA | CTGCACAGGGACAGTGAGAG |
| IAV-NP | CAGGTACTGGGCTATAAGGAC | GCATTGTCTCCGAAGAAATAAG |
| GBP1 | CTAGCTGGGCCGCTAACTC | CTGATCTGGGGAACAACACCC |
| GBP2 | GGTAGGATTTGCCTGTGCGA | AAGCACAGACAAGAGAACAATGC |
| GBP3 | TCTGGATTCGCCACCAGTTC | CGGCTGGACTTTCAGCTTCTA |
| GBP4 | TTCTGGATAACCTGGTGTGGG | GGCGATTCAAAGGCAGAACG |
| GBP5 | AGCCTGTTCCTGCATCTGTT | TGCACCATCCCATTTGTGGAA |
| GBP6 | GCCAACCTAGAAGAGCCTGC | CTACTCTGGACAGAGGAACGC |
| GBP7 | TAGAGGCCCACAATTGCCAC | TTGTTCCAGAGCCCATGAA |
| NEAT1 | GTGGCTGTTGGAGTCGGTAT | TAACAAACCACGGTCCATGA |
| LRRC8B | GGGATCAAGGAAGGACAGGTAAT | TGCTGGTCAACTGGAACCTC |
|  |  |  |

**Table S2.** **Primers used for ChIP-qPCR.**

| **Region** | **Forward primer (5’→3’)** | **Reverse primer (5’→3’)** |
| --- | --- | --- |
| 1 | CAGGGGGATGGGAGTTTTAT | GGGCAGCTGTTTGAGAAGAC |
| 2 | CAGATTGCAAAAACCACACG | TGGAGAGTCATCCTGCGTAA |
| 3 | CAAATTGTTGCCTGGACAGA | AGGAGTGGAAGAGTGGAGCA |
| 4 | GACTGCAAACCAAGTGAACTGA | ATTGAGTCAGTTTGAGAATCCTAGA |
| 5 | AACATGATGCCTCCTCATTCTAGG | GTACTTCATTCAAGTCCACTACCAT |
|  |  |  |

**Table S3. 33 RNA-binding proteins pulled down by GBP1P1.**

| **Gene Symbol** | **Description** | **Score** | **Matches** | **Sequences** |
| --- | --- | --- | --- | --- |
| DHX9 | ATP-dependent RNA helicase A OS=Homo sapiens OX=9606 GN=DHX9 PE=1 SV=4 | 1027 | 76 | 32 |
| ILF3 | Interleukin enhancer binding factor 3, 90kDa, isoform CRA_d OS=Homo sapiens OX=9606 GN=ILF3 PE=4 SV=1 | 277 | 16 | 13 |
| MYH9 | Myosin, heavy polypeptide 9, non-muscle, isoform CRA_a OS=Homo sapiens OX=9606 GN=MYH9 PE=3 SV=1 | 227 | 15 | 14 |
| SART3 | Squamous cell carcinoma antigen recognized by T-cells 3 OS=Homo sapiens OX=9606 GN=SART3 PE=1 SV=1 | 209 | 20 | 16 |
| HNRNPU | Heterogeneous nuclear ribonucleoprotein U OS=Homo sapiens OX=9606 GN=HNRNPU PE=1 SV=1 | 187 | 15 | 11 |
| EIF3A | Eukaryotic translation initiation factor 3 subunit A OS=Homo sapiens OX=9606 GN=EIF3A PE=2 SV=1 | 182 | 21 | 20 |
| CLTC | Clathrin heavy chain OS=Homo sapiens OX=9606 GN=CLTC PE=1 SV=1 | 153 | 12 | 11 |
| CAPRIN1 | Caprin-1 (Fragment) OS=Homo sapiens OX=9606 GN=CAPRIN1 PE=1 SV=1 | 151 | 6 | 5 |
| MAP4 | Microtubule-associated protein 4 OS=Homo sapiens OX=9606 GN=MAP4 PE=1 SV=3 | 135 | 8 | 8 |
| SRRT | Serrate RNA effector molecule homolog OS=Homo sapiens OX=9606 GN=SRRT PE=1 SV=1 | 131 | 12 | 11 |
| NCL | Nucleolin, isoform CRA_b OS=Homo sapiens OX=9606 GN=NCL PE=4 SV=1 | 89 | 4 | 4 |
| ATXN2L | Ataxin 2-like, isoform CRA_a OS=Homo sapiens OX=9606 GN=ATXN2L PE=4 SV=1 | 89 | 6 | 6 |
| FLNA | Filamin-A OS=Homo sapiens OX=9606 GN=FLNA PE=1 SV=1 | 85 | 9 | 9 |
| CCAR1 | Cell division cycle and apoptosis regulator protein 1 (Fragment) OS=Homo sapiens OX=9606 GN=CCAR1 PE=1 SV=1 | 75 | 6 | 6 |
| HNRNPUL2 | Heterogeneous nuclear ribonucleoprotein U-like protein 2 OS=Homo sapiens OX=9606 GN=HNRNPUL2 PE=1 SV=1 | 69 | 10 | 9 |
| FLNB | Filamin-B OS=Homo sapiens OX=9606 GN=FLNB PE=1 SV=2 | 63 | 7 | 7 |
| FAM120A | Constitutive coactivator of PPAR-gamma-like protein 1 OS=Homo sapiens OX=9606 GN=FAM120A PE=1 SV=2 | 57 | 4 | 4 |
| UPF1 | UPF1 OS=Homo sapiens OX=9606 GN=UPF1 PE=2 SV=1 | 56 | 4 | 4 |
| PLEC | Plectin OS=Homo sapiens OX=9606 GN=PLEC PE=1 SV=9 | 56 | 11 | 11 |
| MATR3 | Matrin-3 OS=Homo sapiens OX=9606 GN=MATR3 PE=1 SV=1 | 54 | 2 | 2 |
| SF3A1 | Splicing factor 3a, subunit 1, 120kDa, isoform CRA_a OS=Homo sapiens OX=9606 GN=SF3A1 PE=4 SV=1 | 53 | 3 | 3 |
| RRBP1 | Ribosome-binding protein 1 OS=Homo sapiens OX=9606 GN=RRBP1 PE=1 SV=1 | 52 | 4 | 4 |
| DHX29 | ATP-dependent RNA helicase DHX29 OS=Homo sapiens OX=9606 GN=DHX29 PE=1 SV=1 | 49 | 3 | 3 |
| EIF5B | Eukaryotic translation initiation factor 5B OS=Homo sapiens OX=9606 GN=EIF5B PE=1 SV=1 | 46 | 5 | 5 |
| PRRC2C | Protein PRRC2C (Fragment) OS=Homo sapiens OX=9606 GN=PRRC2C PE=1 SV=1 | 40 | 2 | 2 |
| TCOF1 | Treacle protein (Fragment) OS=Homo sapiens OX=9606 GN=TCOF1 PE=1 SV=1 | 36 | 1 | 1 |
| SF3B2 | Splicing factor 3B subunit 2 OS=Homo sapiens OX=9606 GN=SF3B2 PE=1 SV=2 | 35 | 4 | 4 |
| DSP | Desmoplakin OS=Homo sapiens OX=9606 GN=DSP PE=1 SV=3 | 34 | 6 | 6 |
| KTN1 | Kinectin 1 (Kinesin receptor), isoform CRA_a OS=Homo sapiens OX=9606 GN=KTN1 PE=4 SV=1 | 33 | 4 | 4 |
| ZFR | Zinc finger RNA binding protein OS=Homo sapiens OX=9606 GN=ZFR PE=2 SV=1 | 31 | 3 | 3 |
| SUPT16H | FACT complex subunit SPT16 OS=Homo sapiens OX=9606 GN=SUPT16H PE=1 SV=1 | 29 | 2 | 2 |
| RBM10 | RNA binding motif protein 10 isoform 2 (Fragment) OS=Homo sapiens OX=9606 GN=RBM10 PE=2 SV=1 | 26 | 2 | 2 |
| ACIN1 | Apoptotic chromatin condensation inducer in the nucleus OS=Homo sapiens OX=9606 GN=ACIN1 PE=1 SV=1 | 23 | 5 | 4 |
